# Proteomic and Metabolomic Analysis of Bone Marrow and Plasma from Patients with Extramedullary Multiple Myeloma Identifies Distinct Protein and Metabolite Signatures

**DOI:** 10.3390/cancers15153764

**Published:** 2023-07-25

**Authors:** Katie Dunphy, Despina Bazou, Michael Henry, Paula Meleady, Juho J. Miettinen, Caroline A. Heckman, Paul Dowling, Peter O’Gorman

**Affiliations:** 1 Department of Biology, Maynooth University, W23 F2K8 Kildare, Ireland; paul.dowlling@mu.ie; 2 Department of Haematology, Mater Misericordiae University Hospital, D07 AX57 Dublin, Ireland; despina.bazou@ucd.ie (D.B.); pogorman@mirtireland.com (P.O.); 3 National Institute for Cellular Biotechnology, Dublin City University, D09 NR58 Dublin, Ireland; michael.henry@dcu.ie (M.H.); paula.meleady@dcu.ie (P.M.); 4 Institute for Molecular Medicine Finland-FIMM, HiLIFE–Helsinki Institute of Life Science, iCAN Digital Precision Cancer Medicine Flagship, University of Helsinki, 00290 Helsinki, Finland; juho.miettinen@helsinki.fi (J.J.M.); caroline.heckman@helsinki.fi (C.A.H.)

**Keywords:** multiple myeloma, extramedullary multiple myeloma, extramedullary disease, proteomics, mass spectrometry, clinical proteomics, metabolomics

## Abstract

**Simple Summary:**

Extramedullary multiple myeloma (EMM) is a rare and aggressive subtype of multiple myeloma which is associated with a poor prognosis. Here, we used mass spectrometry to illustrate that extramedullary multiple myeloma patients have a bone marrow and plasma protein signature that is distinct from multiple myeloma patients without extramedullary spread. We used bioinformatic tools to analyse differentially expressed proteins and verified the increased abundance of three proteins (VCAM1, HGFA, PEDF) in the plasma of patients with EMM. Considering the paucity of informative biomarkers and effective therapeutic approaches for the treatment of EMM, this study may provide direction for the discovery of novel diagnostic and therapeutic approaches and markers of extramedullary progression.

**Abstract:**

Multiple myeloma (MM) is an incurable haematological malignancy of plasma cells in the bone marrow. In rare cases, an aggressive form of MM called extramedullary multiple myeloma (EMM) develops, where myeloma cells enter the bloodstream and colonise distal organs or soft tissues. This variant is associated with refractoriness to conventional therapies and a short overall survival. The molecular mechanisms associated with EMM are not yet fully understood. Here, we analysed the proteome of bone marrow mononuclear cells and blood plasma from eight patients (one serial sample) with EMM and eight patients without extramedullary spread. The patients with EMM had a significantly reduced overall survival with a median survival of 19 months. Label-free mass spectrometry revealed 225 proteins with a significant differential abundance between bone marrow mononuclear cells (BMNCs) isolated from patients with MM and EMM. This plasma proteomics analysis identified 22 proteins with a significant differential abundance. Three proteins, namely vascular cell adhesion molecule 1 (VCAM1), pigment epithelium derived factor (PEDF), and hepatocyte growth factor activator (HGFA), were verified as the promising markers of EMM, with the combined protein panel showing excellent accuracy in distinguishing EMM patients from MM patients. Metabolomic analysis revealed a distinct metabolite signature in EMM patient plasma compared to MM patient plasma. The results provide much needed insight into the phenotypic profile of EMM and in identifying promising plasma-derived markers of EMM that may inform novel drug development strategies.

## 1. Introduction

Multiple myeloma (MM) is characterised by the uncontrolled proliferation of plasma cells in the bone marrow, resulting in the production of large amounts of non-functional monoclonal antibodies or paraproteins, which can be detected in the blood or urine [1]. MM is the second most common blood cancer and accounts for approximately 1% of all cancers [2]. The approval of various therapeutics, including proteasome inhibitors, immunomodulatory drugs, and monoclonal antibodies, have improved the overall survival of MM patients over the last two decades. However, despite the introduction of these novel therapeutics, MM remains an incurable disease due to the development of drug resistance and repeated relapses [3]. 

As outlined in the Revised International Staging System (RISS), multiple factors, including tumour burden, serum biomarker levels and the presence of high-risk cytogenetics, help define MM prognosis [4]. One poor prognostic factor is the presence of extramedullary multiple myeloma (EMM), an aggressive manifestation of MM where clonal plasma cells become independent of the bone marrow microenvironment (BME) and colonise distal organs and soft tissues outside of the bone marrow, such as the skin, liver and lungs [5]. EMM can be detected at diagnosis or relapse, both of which indicate poor prognosis. There is no consensus on the median survival of EMM patients; however, several studies have reported significantly reduced median survival in newly diagnosed and relapsed MM patients with extramedullary disease compared to those without plasmacytomas [6,7,8]. The reported incidence of EMM varies between studies, ranging from 0.5–4.8% in newly diagnosed MM and 3.4–14% in relapsed/refractory MM [8].

Treatment resistance is commonly associated with EMM [9,10]. When considering treatment options, EMM is treated aggressively, similarly to how high-risk MM is treated. MM is still treated empirically with conventional myeloma therapies, meaning there is a lack of targeted therapeutic options for MM variants, such as EMM. The low number of prospective studies specifically focusing on EMM impedes a clinician’s ability to make strong treatment recommendations [8]. For this reason, several studies have advised future large MM-focused clinical trials to evaluate EMM patients as a defined subgroup to inform therapeutic decision making [8,11]. There is therefore a need for novel therapeutic targets and treatment strategies to improve the survival outcome for EMM patients.

Despite the aggressiveness of EMM, the molecular mechanisms that contribute to the escape of malignant plasma cells from the bone marrow and the colonization of distant tissues are poorly understood [5]. Several studies have reported high-risk genetic abnormalities, such as del(17p13), and dysregulated cell adhesion and migratory pathways, as contributing to extramedullary progression [12,13]. The increased expression of the chemokine receptor CXCR4 has been linked to the development of an epithelial-to-mesenchymal-like phenotype that is associated with medullary and extramedullary metastasis [14,15]. Furthermore, myeloma cells derived from extramedullary sites are often plasmablastic, a morphological trait associated with more aggressive disease [16,17]. Dissecting the key signalling pathways that facilitate the survival and proliferation of plasma cells outside of the bone marrow is crucial for identifying novel therapeutic targets in EMM. Furthermore, identifying clinically relevant non-invasive markers of EMM may facilitate the early detection of extramedullary lesions. 

In this study, we performed a mass-spectrometry (MS)-based pilot proteomic analysis of matched bone marrow mononuclear cells (BMNCs) and blood plasma from MM patients with and without extramedullary spread (*n* = 17). A targeted metabolomic analysis of blood plasma was also performed to explore the metabolic signature of EMM plasma. Through these analyses, we aimed to (i) identify biological processes or signalling pathways associated with extramedullary transition, (ii) identify potential therapeutic targets and prognostic markers for further investigation and (iii) reveal plasma-based biomarkers for diagnostic and/or prognostic clinical use. Collectively, this study contributes to the current understanding of the molecular phenotype associated with EMM. 

## 2. Materials and Methods

### 2.1. Patient Information and Sample Collection

Bone marrow mononuclear cells (BMNCs) and blood EDTA plasma samples from age- and gender-matched MM (*n* = 8) and EMM patients (*n* = 9, 1 serial sample) were obtained from the Finnish Hematology Registry and Clinical Biobank (FHRB). In this research paper, EMM refers to patients with soft tissue plasmacytomas outside of the bone marrow and not paraskeletal plasmacytomas [18]. Patient characteristics are summarised in Table 1. Cytogenetic information was also recorded (Figure S1). Sample collection, with informed consent, took place between 2013 and 2020 across several Finnish university hospitals and other haematology units. Median age was 65. Samples were obtained from 4 female and 12 male subjects. To analyse the association of the plasma-based markers of EMM with drug resistance, EDTA plasma was obtained from a second cohort of patients (*n* = 44) stratified based on ex vivo drug sensitivity resistance testing (DSRT) performed on CD138+ myeloma cells isolated from bone marrow aspirates. These plasma samples from 44 MM patients with corresponding DSRT data were also obtained from the FHRB. The FHRB is authorised and approved by the Finnish National Supervisory Authority for Welfare and Health (Valvira) and Finnish National Medical Ethics Committee, respectively. Samples were stored at −80 °C.

### 2.2. Bone Marrow Mononuclear Cells Sample Preparation

Cryopreserved BMNCs were thawed in a 37 °C water bath. BMNCs were isolated and washed twice with phosphate-buffered saline (PBS). The supernatant was removed, and cell pellets were solubilised in 200 μL of lysis buffer (4% SDS, 100 mM Tris/HCl pH 7.6, 0.1 M DTT, protease inhibitors). Protein quantitation was performed using the Pierce™ 660 nm protein assay (Thermo Fisher Scientific, Waltham, MA, USA), as described by the manufacturer’s guidelines. Filter-aided sample preparation (FASP) was applied for proteolytic digestion [19]. A total of 15 μg of protein from each sample was digested. Briefly, samples were subject to a series of centrifugal steps using 8 M urea and 50 mM iodoacetamide to facilitate detergent removal, buffer exchange and protein alkylation. Overnight trypsin digestion was carried out using a 1:25 enzyme-to-protein ratio in 50 mM ammonium bicarbonate digestion buffer. The tryptic peptides were acidified at a 1:10 ratio using 2% TFA and 20% ACN.

### 2.3. Label-Free Liquid Chromatography—Tandem Mass Spectrometry Analysis of BMNCs

Liquid chromatography tandem mass spectrometry (LC-MS/MS) was performed using the Thermo UltiMate 3000 nano system and directly coupled in-line with the Thermo Orbitrap Fusion Tribrid mass spectrometer. The maximum loading amount (~800 ng) was loaded for mass spectrometry analysis. PepMap100 (C18, 300 µm × 5 mm) and Acclaim PepMap 100 (75 µm × 50 cm, 3 µm bead diameter) columns were used as the trapping and analytical columns, respectively. Peptides were eluted over the following binary gradient: LC Solvent A and LC Solvent B using 2–32% Solvent B for 75 min, 32–90% Solvent B for 5 min and holding at 90% for 5 min at a flow rate of 300 nL/min. A data-dependent acquisition strategy was applied with full MS scans in the 380–1500 *m*/*z* range with a resolution of 120,000 at 200 *m*/*z*. A top-speed approach with a cycle time of 3 s was used for tandem MS analysis, with selected precursor ions isolated with an isolation width of 1.6 Da. The intensity threshold for fragmentation was set to 5000 and included peptides with charge states of 2+ to 7+. A higher energy collision dissociation (HCD) approach was applied with a normalised collision energy of 28% and tandem MS spectra were acquired in the linear ion trap with a fixed first *m*/*z* of 110, and a dynamic exclusion of 50 s was applied. A targeted automatic gain control (AGC) was set to 2 × 104 with a maximum injection time set at 35 ms.

### 2.4. Data Analysis of BMNCs Mass Spectrometry Results

The UniProtKB-SwissProt Homo Sapiens database with Proteome Discoverer 2.2 using Sequest HT (Thermo Fisher Scientific) and a percolator were used for the identification of peptides and proteins. Search parameters were set as follows: (i) MS/MS mass tolerance was set to 0.02 Da, (ii) peptide mass tolerance was set at 10 ppm, (iii) variable modifications included methionine oxidation, (iv) fixed modification settings for carbamido-methylation and (v) tolerance for up to two missed cleavages. Peptide probability was set to high confidence, and a minimum XCorr score of 1.5 for 1, 2.0 for 2, 2.25 for 3 and 2.5 for 4 charge states was applied for peptide filtering. The associated label-free quantitation software Progenesis QI for Proteomics (version 2.0; Nonlinear Dynamics, a Waters company, Newcastle upon Tyne, UK) was used for quantitative data analysis. Datasets were imported into Progenesis QI software. Protein identifications were deemed to be differentially expressed when specific criteria were met. Missing values were imputed using a width of 0.3 and down shift of 1.8 to enable statistical comparisons. The criteria were: ANOVA *p*-value of ≤0.05 between experimental groups, fold change ≥1.5 between experimental groups, proteins with ≥2 unique peptides contributing to the identification, and quantification data in >60% of samples. Pathway enrichment and gene ontology (GO) enrichment analysis was performed by submitting Uniprot accession IDs to the g:Profiler online bioinformatics tool (https://biit.cs.ut.ee/gprofiler/gost) (accessed on 24 November 2022) [20]. Term size was set to between 5 and 2000. 

### 2.5. Gene Expression Analysis Using the CoMMpass Dataset

To determine the association of the most differentially abundant proteins with MM prognosis, we used the mRNA expression data from the MMRF CoMMpass study. The gene expression profiles and survival data of patients with MM (*n* = 784) were obtained and analysed using UCSC Xena (https://xena.ucsc.edu/) (accessed on 17 November 2022). Raw count values and clinical data were downloaded from the Xena website and normalised using the R package “deseq2”. Survival analysis was performed using the “survival” and “RegParallel” packages and survival curves were illustrated using the Kaplan–Meier method. Proteins significantly changed between EMM BMNCs and MM BMNCs were analysed to identify the prognostic relevance of the gene expression of these proteins. Median expression values were used to binarise the genes. Gene expression results with log-rank *p*-values < 0.05 were considered significantly associated with MM survival.

### 2.6. Blood Plasma Sample Preparation

High abundant plasma proteins were depleted using the Proteome Purify 12 Human Serum Protein Immunodepletion Resin (R&D Systems, Minneapolis, MN, USA). Briefly, 10 μL of plasma was mixed with 1 mL of immunodepletion resin for 60 min. The mixture was transferred to Spin-X filter units and centrifuged. The protein concentration of the resulting eluate was determined using the Pierce™ 660 nm protein assay (Thermo Fisher Scientific). Protein digestion was performed using the FASP protocol, as described above. A total of 10 μg of protein was digested at a 1:25 enzyme-to-protein ratio. The tryptic digest was acidified at a 1:10 ratio using 2% TFA, 20% ACN.

### 2.7. Label-Free Liquid Chromatography-Tandem Mass Spectrometry Analysis of Plasma

LC-MS/MS was performed using the Ultimate 3000 NanoLC system (Dionex Corporation, Sunnyvale, CA, USA) coupled with a Q-Exactive mass spectrometer (Thermo Fisher Scientific). A total of 14 μL, containing ~1μg of digested protein was loaded. Samples were loaded onto a C18 trap column (C18 PepMap, 300 µm id × 5 mm, 5 µm particle size, 100 Å pore size; Thermo Fisher Scientific) and resolved on an analytical Biobasic C18 Picofrit column (C18 PepMap, 75 µm id × 50 cm, 2 µm particle size, 100 Å pore size; Dionex). Peptides generated were eluted over a 120 min gradient. The Q-Exactive was operated in positive, data-dependent acquisition (DDA) mode and externally calibrated. Full-scan spectra were collected at a fixed resolution of 140,000 and a mass range of 300–1700 *m*/*z*. Fragmentation spectra were acquired through the collision-induced dissociation (CID) of the fifteen most intense ions per scan, at a resolution of 17,500 and range of 200–2000 *m*/*z*. A dynamic exclusion window was applied within 30 s.

### 2.8. Data Analysis of Plasma Mass Spectrometry Results

Raw files from the mass spectrometry analysis were searched using the associated software, Proteome Discoverer 2.5 (Thermo Fisher Scientific). Protein identification and label-free quantitation (LFQ) was performed. The resulting dataset was imported into Perseus (1.6.14.0). Proteins with ≥2 peptides contributing to the identification and quantitative values in >70% samples were retained for downstream analysis. Missing values were imputed using a width of 0.3 and a down-shift of 1.8 to enable statistical comparisons. Statistically significant differentially abundant proteins were identified based on a false discovery rate (FDR)-adjusted *p*-value < 0.1 and fold change >1.5 between experimental groups.

### 2.9. Enzyme-Linked Immunosorbent Assays (ELISAs)

The concentrations of six proteins (vascular cell adhesion protein 1 (VCAM1), aminopeptidase N (CD13), butyrylcholinesterase (BCHE), hepatocyte growth factor activator (HGFA), alpha 2-macroglobulin (A2M) and pigment epithelium-derived factor (PEDF)) in blood plasma were measured by ELISA (DuoSet ELISA kits, R&D Systems). The following plasma dilutions were used: VCAM1 (1:1500), CD13 (1:75), BCHE (1:2000), HGFA (1:2000), A2M (1:100,000) and PEDF (1:8000). The plasma concentrations of VCAM1, PEDF and HGFA, at the same dilutions, were also analysed in the second MM patient cohort (*n* = 44).

### 2.10. Statistical Analysis

The statistical analysis of ELISA results, receiver-operating characteristic (ROC) curve analysis and correlation analyses were performed using Graphpad Prism (8.0.2.263) and MedCalc (version 20.118). Parametric *t*-tests were used to evaluate statistical significance. Outliers were removed using the ROUT method (Q = 1%). ROC curve analysis was used to determine the discriminatory performance of the verified statistically significant differentially abundant (SSDA) plasma proteins. The ROC curves evaluated the specificity (false positive fraction) and sensitivity (true positive fraction) of the potential protein biomarkers. Optimal cut-off points were selected using Youden’s index. The area under the curve (AUC) was calculated to summarise the accuracy of the classification. Logistic regression analysis was performed using MedCalc.

### 2.11. Targeted Metabolomic Analysis

The targeted metabolomic analysis of medullary MM and EMM blood plasma samples was performed using the MxP^®^ Quant 500 kit (Biocrates Life Sciences AG, Innsbruck, Austria) with a SCIEX QTRAP 6500plus mass spectrometer. The MxP^®^ Quant 500 kit is capable of quantifying more than 600 metabolites from 26 compound classes. Quality control (QC) samples were employed to monitor the performance of the analysis with metabolite concentration in each sample normalised based on these QC samples. Isotopically labelled internal standards and seven-point calibration curves were used in the quantitation of amino acids and biogenic amines. The semi-quantitative analysis of other metabolites was performed using internal standards. Data quality was evaluated by checking the accuracy and reproducibility of QC samples. Metabolites were included only when the concentrations of the metabolites were above the limit of detection (LOD) in >75% of plasma samples. Data were imported into MetaboAnalyst 5.0 for further analysis. Feature filtering was performed based on relative standard deviation (RSD) and the resulting data were log-transformed and autoscaled. Metabolites of interest were identified based on *p*-value < 0.05 and fold-change >1.2 between experimental groups. Supervised statistical approaches were used to further interrogate the data.

## 3. Results

### 3.1. Clinical Information

Eight samples with MM but without extramedullary spread and nine samples with EMM were included in this study. Clinical data was obtained and summarised (Table 1, Figure S1). The median age was 65. Six males and two females were included in each group. Overall survival (OS) was statistically significantly decreased in patients with EMM compared to MM patients without extramedullary spread (Log-rank = 3.977, *p* = 0.046) (Figure S2). The median OS of patients with EMM and those without extramedullary spread was 19 months and 83 months, respectively.

### 3.2. Identification of Differentially Abundant Proteins in the Bone Marrow of MM Patients with and without Extramedullary Spread

**Figure 1:**
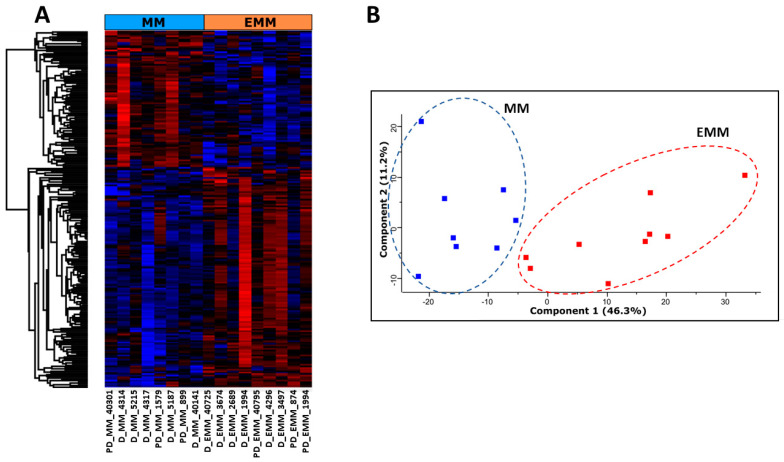
Proteomic profile of BMNCs from EMM patients and medullary MM patients. (**A**) Hierarchical clustering analysis of the statistically significant differentially abundant (SSDA) proteins between MM and EMM groups. The colours from blue to red represent the relative protein levels between the two groups. (**B**) Principal component analysis (PCA) illustrating a clear distinction between MM patients with EMM and those without. Each dot represents a patient sample with EMM samples highlighted in red and MM samples highlighted in blue.

To examine proteomic changes in the bone marrow of MM patients with and without EMM, BMNCs were isolated and proteolytically digested. Nine EMM samples—including one serial sample—and eight MM without extramedullary spread samples were analysed using LC-MS/MS. A total of 4589 proteins and 225 significantly differentially abundant proteins were identified based on ANOVA corrected *p*-value < 0.05 and fold change >1.5 (Figure 1, Table S1). Of these, 139 proteins were increased in abundance and 86 proteins were decreased in abundance in EMM BMNCs compared to MM BMNCs (Table 2 and Table 3). The hierarchical clustering of protein abundance and principal component analysis (PCA) demonstrated a clear change in the proteomic profile of mononuclear cells from MM patients with extramedullary spread and those without (Figure 1A,B).

**Figure 2:**
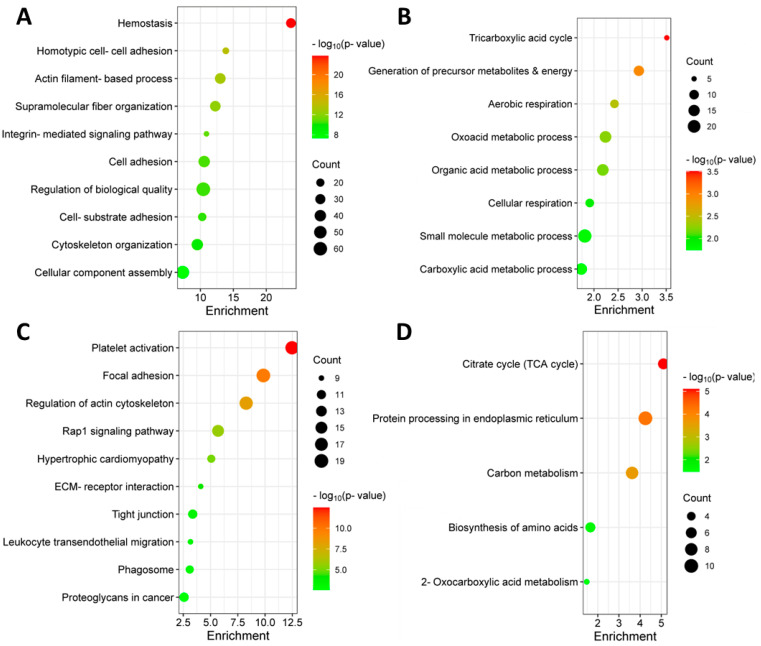
Gene ontology (GO) and Kyoto Encyclopedia of Genes and Genomes (KEGG) functional enrichment analysis of SSDA proteins. (**A**) Bubble plot of GO gene set enrichment analysis (biological processes) of proteins increased in abundance in EMM. (**B**) Bubble plot of GO gene set enrichment analysis (biological processes) of proteins decreased in abundance in EMM. (**C**) Bubble plot of KEGG pathway enrichment analysis of proteins increased in abundance in EMM. (**D**) Bubble plot of KEGG pathway enrichment analysis of proteins decreased in abundance in EMM. Enrichment value = −log_10_(*p*-value).

Proteins found to be increased or decreased in abundance in EMM were characterised based on gene ontology enrichment and KEGG pathway enrichment (Figure 2). Proteins increased in abundance in EMM mononuclear cells were associated with migratory pathways, including focal adhesion, tight junction, Rap1 signalling pathway and leukocyte endothelial migration. Interestingly, proteins decreased in abundance in EMM BMNCs were associated with various metabolic pathways, including the tricarboxylic acid (TCA) cycle, suggesting a possible metabolic change in the cells of the bone marrow microenvironment during EMM transition [21].

### 3.3. Association of Gene Expression with Prognosis Using the MMRF CoMMpass Study Data

**Figure 3:**
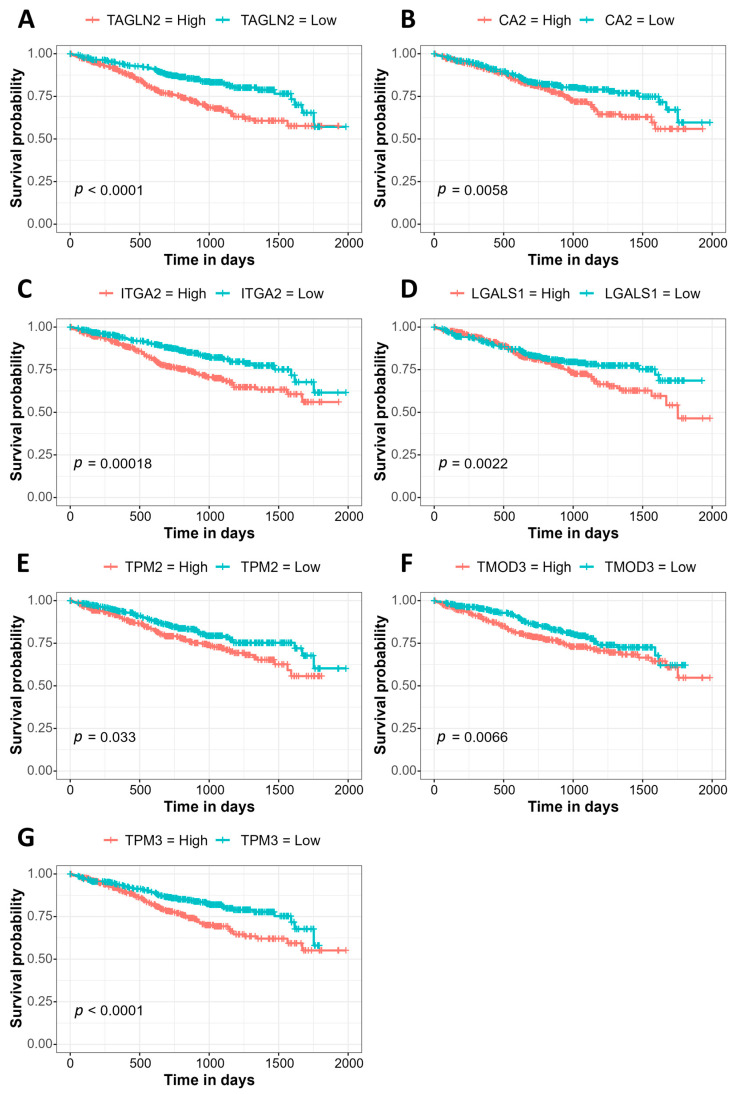
Kaplan–Meier curves illustrating genes whose expression (high/low) is significantly associated with survival in MM using the MMRF CoMMpass RNASeq dataset. (**A**) TAGLN2, (**B**) CA2, (**C**) ITGA2, (**D**) LGALS1, (**E**) TPM2, (**F**) TMOD3, (**G**) TPM3.

To determine whether the proteins most significantly increased in abundance in EMM mononuclear cells were associated with a poor prognosis in MM, we performed a Kaplan–Meier gene expression analysis on the 25 proteins most significantly increased in abundance in EMM BMNCs using the MMRF CoMMpass dataset (Table 2). The increased expression of seven genes was associated with a significantly worse prognosis in MM. These included genes associated with focal adhesion and actin regulation, transgelin 2 (TAGLN2), integrin alpha 2 (ITGA2), tropomyosin beta chain (TPM2) and tropomyosin alpha-3 chain (TPM3), as well as carbonic anhydrase 2 (CA2), galectin-1 (LGALS1) and tropomodulin-3 (TMOD3) (Figure 3). For our cohort, we divided the samples into high and low expression groups for each of the seven biomarkers. Survival analysis revealed a trend towards decreased overall survival in those with the high expression of six (TAGLN2, CA2, ITGA2, LGALS1, TPM2, TMOD3) out of the seven proteins analysed. The high expression of TMOD3 was significantly associated with a poorer overall survival compared to those with a low expression of TMOD3 (Figure S3). Thus, the increased abundance of these proteins is associated with the aggressive EMM phenotype, as well as poorer overall survival in MM. 

### 3.4. Identification of Significantly Differentially Abundant Proteins in the Plasma of MM Patients with and without Extramedullary Spread

**Figure 4:**
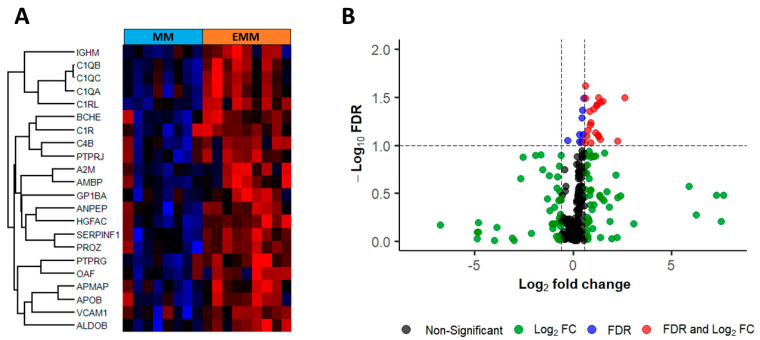
Heatmap and volcano plot of statistically significant differentially abundant (SSDA) proteins identified in the label-free mass spectrometry analysis of blood plasma. (**A**) Hierarchical clustering of SSDA proteins. The colours from blue to red represent the relative protein levels between the two groups. (**B**) Volcano plot of SSDA proteins. Red points represent proteins significantly increased in abundance in EMM plasma. Green points indicate proteins with a log2 fold change >1.5 but false discovery rate (FDR) *p*-value > 0.1. Blue points indicate proteins with an FDR *p*-value < 0.1 but log2 fold change <1.5. Black points indicate proteins with no significant difference.

Matched blood plasma samples from MM (*n* = 8) and EMM (*n* = 9, 1 serial sample) patients, taken on the same date as the BMNCs, were analysed through label-free LC-MS/MS to identify changes in the plasma proteome of patients with and without extramedullary lesions. A total of 524 proteins and 22 significantly differentially abundant proteins were identified based on FDR corrected *p*-value < 0.1 and fold change >1.5 (Figure 4, Table 4). All significant proteins were increased in abundance in EMM plasma samples compared to MM patient plasma without extramedullary spread. Only one protein, platelet glycoprotein Ib alpha chain (GP1BA), was differentially expressed in both BMNCs and blood plasma.

### 3.5. Verification of Differentially Expressed Plasma Proteins Identified by LC-MS/MS

**Figure 5:**
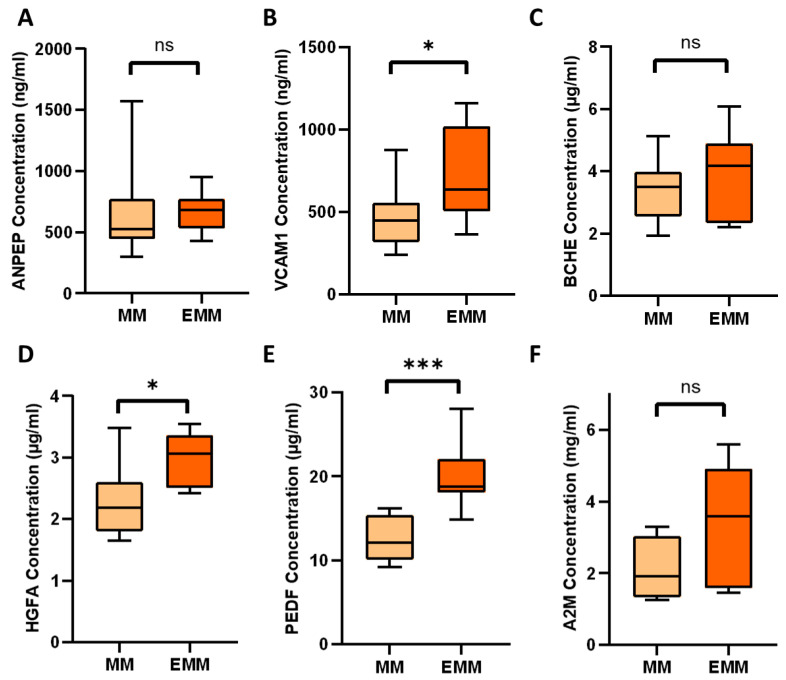
Plasma levels of SSDA proteins measured by ELISA. (**A**) ANPEP, (**B**) VCAM1, (**C**) BCHE, (**D**) HGFA, (**E**) PEDF and (**F**) A2M plasma levels in the EMM and medullary MM groups. Significance is marked as follows: ns ‘not significant’, *p* ≤ 0.05 ‘*’, *p* ≤ 0.001 ‘***’.

**Figure 6:**
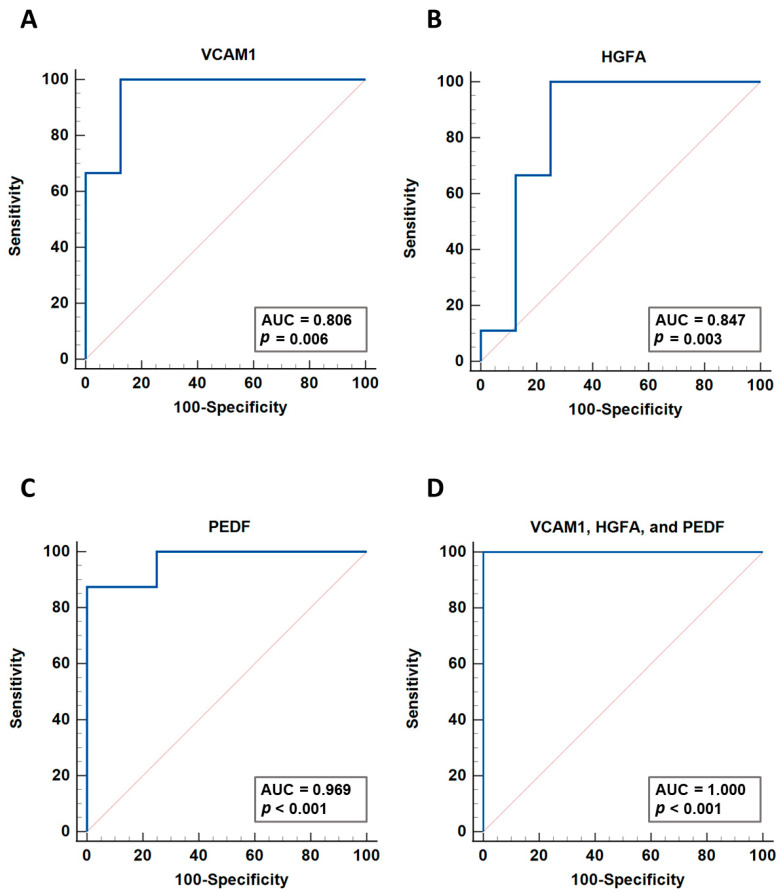
ROC curve analysis of three potential biomarkers. (**A**) VCAM1, (**B**) HGFA and (**C**) PEDF ROC curves. (**D**) Logistic regression analysis with a ROC curve illustrating the discriminatory power of combining VCAM1, HGFA and VCAM1.

Six proteins found to be increased in abundance in the blood plasma of EMM patients were verified via ELISA: ANPEP, VCAM1, BCHE, HGFA, PEDF and A2M. Three of the six proteins analysed (VCAM1, HGFA, PEDF) were verified as being significantly increased in abundance in EMM plasma (Figure 5). Although ANPEP, BCHE and A2M did not reach statistical significance, we observed trends towards increased abundance in EMM plasma, which would warrant further investigation in a larger cohort of samples. To explore the potential of the three verified proteins as biomarkers, we performed individual ROC curve analyses and a multivariate analysis of the biomarker combination. ROC curves were constructed and the area under the curve (AUC) values were calculated (Figure 6). VCAM1, HGFA and PEDF were found to have good prediction ability for EMM with AUC values of 0.806, 0.847 and 0.969, respectively. The combination of these biomarkers using a logistic regression analysis resulted in a larger AUC value of 1 and 95% confidence interval of 0.794–1. 

### 3.6. VCAM1 Plasma Concentrations Are Increased in Patients Most Sensitive to the BCL-2 Inhibitors, Venetoclax and Navitoclax

**Figure 7:**
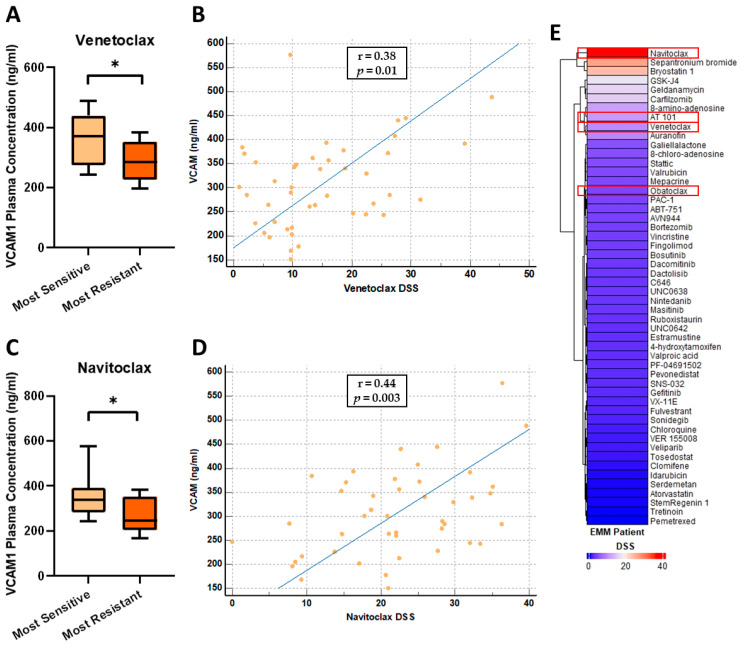
Plasma concentration of VCAM1 in patients most sensitive and most resistant to the BCL-2 inhibitors (**A**) venetoclax and (**C**) navitoclax. (**B**) Correlation between VCAM1 plasma concentration and venetoclax sensitivity. (**D**) Correlation between VCAM1 plasma concentration and navitoclax sensitivity. (**E**) Heatmap illustrating the varying DSS scores of an EMM patient. Drugs with DSS = 0 were removed from this figure. Drugs from the BCL2 inhibitor drug family are highlighted by the red boxes. Significance is marked as follows: *p* ≤ 0.05 ‘*’.

As EMM is often associated with drug resistance, we tested the levels of VCAM1, HGFA and PEDF in the plasma of patients whose CD138+ myeloma cells had been evaluated by ex vivo drug sensitivity resistance testing (DSRT) (*n* = 44) [22,23]. VCAM1, HGFA, and PEDF plasma concentrations in this independent set of MM samples (*n* = 43, excluding 1 EMM sample) showed a similar pattern as observed in the MM group above. VCAM1 concentrations ranged from 150.9–488.2 ng/mL with a median and mean concentration of 289.7 ng/mL and 301 ng/mL, respectively. HGFA concentrations ranged from 1.039–3.372 µg/mL with a median and mean concentration of 2.155 µg/mL and 2.143 µg/mL, respectively. PEDF concentrations ranged from 8.901–19.22 µg/mL with a median and mean concentration of 12.97 µg/mL and 13.39 µg/mL, respectively. The median concentrations of VCAM1, HGFA and PEDF observed in this MM sample set were considerably lower than the median concentrations observed in the EMM group above (VCAM1 = 634.7 ng/mL, HGFA = 3.068 µg/mL, PEDF = 18.77 µg/mL) supporting our findings that these three proteins are increased in abundance in EMM patient plasma. We used Pearson’s correlation analysis to evaluate whether plasma concentrations of VCAM1, HGFA and PEDF correlated with patient sensitivity to individual drugs based on the individual drug sensitivity scores (DSS). Based on the DSS, the samples were stratified into least sensitive and most sensitive groups to various drugs, including the BCL-2 inhibitors, venetoclax and navitoclax. The level of soluble VCAM1 (sVCAM1) was found to be significantly increased in patients considered most sensitive to venetoclax and navitoclax (Figure 7A,C). In addition, higher levels of sVCAM1 weakly correlated with increased sensitivity to venetoclax and navitoclax (Pearson’s correlation coefficient r = 0.38 (*p* = 0.0116) and r = 0.44 (*p* = 0.0026), respectively) (Figure 7B,D). One patient from this cohort had EMM at the time of sampling and therefore had corresponding DSS values available. As expected, this patient was found to be resistant to many of the drugs tested (Figure 7E). Interestingly, this sample was highly sensitive to navitoclax and demonstrated some sensitivity to the other BCL-2 inhibitors tested, AT 101, venetoclax and obatoclax (Figure 7E).

### 3.7. Targeted Metabolomic Analysis of Blood Plasma from MM Patients with and without Extramedullary Spread

**Figure 8:**
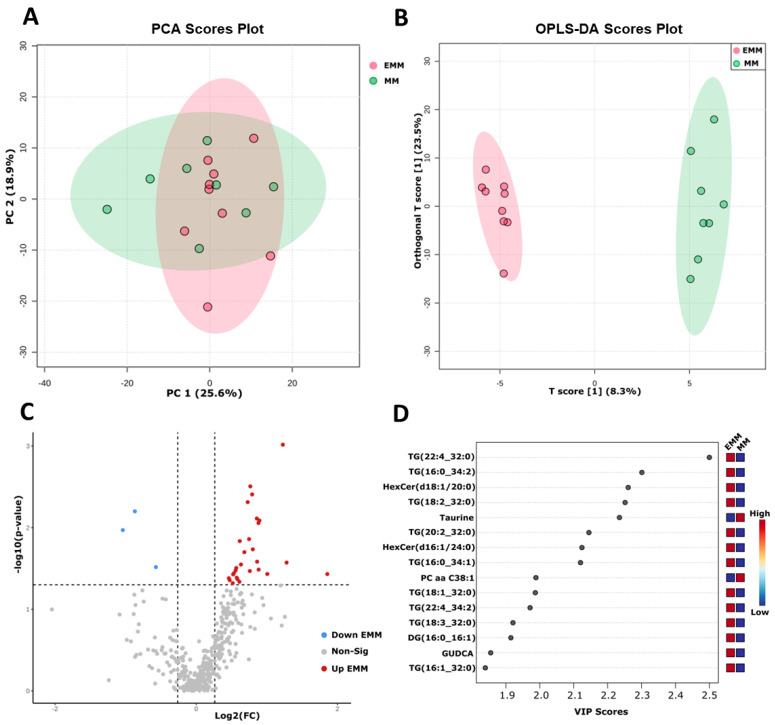
Targeted metabolomic analysis of EMM and MM plasma samples. (**A**) PCA score plot of EMM and MM samples. (**B**) Orthogonal projection to latent structure discriminant analysis (OPLS-DA) scores plot. Cumulative R2X = 0.555, R2Y = 0.99 and Q2 = 0.444. (**C**) Volcano plot identifying significantly altered metabolites in MM patients with and without extramedullary spread (*p*-value ≤ 0.05, FC > 1.2). (**D**) Variable importance in projection (VIP) scores plot depicting the 15 most significant metabolites contributing to the MM group separations observed in the model depicted in (**B**). Red squares indicate metabolites of high abundance in EMM plasma. Blue squares indicate metabolites of low abundance in EMM plasma.

Using a targeted metabolomic/lipidomic technique, we compared the metabolic profile of MM and EMM patient plasma. We applied the unsupervised clustering approach and principal component analysis (PCA); however, no clear separation was observed between the EMM group and the medullary MM group (Figure 8A). A supervised clustering technique termed the orthogonal projection to latent structure discriminant analysis (OPLS-DA) was also used to determine separation between the two groups (Figure 8B). In the OPLS-DA model, R2 refers to the explained variance between the components, whereas Q2 is calculated by full cross validation to indicate the goodness of prediction. R2 and Q2 values closer to 1 indicate a better predictive model. Permutation analysis results (Q2 = 0.444, *p* = 0.042; R2Y = 0.99, *p* = 0.032) demonstrated that the model was not overfitted. In this analysis, the Q2 value of 0.444 indicated weak predictive power; however, due to the heterogeneity of human samples and the small sample size, a Q2 value of >0.4 is acceptable [24,25]. Discriminatory variables responsible for the group separation were identified using the OPLS-DA variable importance in the projection (VIP) score (Figure 8D).

Univariate analysis using a *t*-test identified 31 metabolites with significant differential abundance between the two groups (Figure 8C, Table S2). Of these, 28 metabolites were increased, and 3 metabolites were decreased in EMM plasma. A total of 26 of the 28 metabolites increased in EMM plasma were lipids. The total level of triglycerides was found to be higher in patients with EMM; however, this did not reach significance (*p* = 0.099) (Figure S3). The bile acid, glycoursodeoxycholic acid and the amino acid, tyrosine, were the only non-lipids found to be significantly increased in EMM plasma. We analysed the correlation between the metabolites/lipids and proteins identified in the plasma of EMM patients using Spearman’s rank correlation (Figures S4 and S5). Taurine, phenylalanine betaine and phosphatidylcholine with a diacyl residue sum of C38:1 (PC aa C38:1) were negatively correlated with the proteins, whereas all other metabolites identified were positively correlated with the plasma proteins identified in our proteomics analysis. 

## 4. Discussion

The introduction of novel therapeutics over the last 20 years has significantly improved survival rates of patients with MM. However, the presence of extramedullary lesions remains a poor prognostic indicator and an area of significant unmet need due to the lack of understanding of the history of this entity and the limited treatment options available. Historically, extramedullary spread was considered rare; however, the incidence has risen in recent years mainly due to improved sensitivity of imaging techniques and possible late emergence of EMM clones with the longer OS of MM patients [26]. Currently, EMM is diagnosed using imaging modalities. The International Myeloma Working Group (IMWG) recommends the use of fluorine 18 fluorodeoxyglucose (FDG) PET/CT to detect extramedullary lesions; however, the disadvantages of this imaging technique include the high cost, limited availability and lack of imaging standardization [9,27]. The underlying mechanisms that facilitate the spread and survival of malignant plasma cells outside of the bone marrow microenvironment are poorly understood [28].

This study aimed to characterise the proteome of the bone marrow microenvironment and blood plasma of MM patients with EMM to identify potential biomarkers that could serve as predictors of extramedullary development, prognostic biomarkers of MM and possibly contribute to the identification of novel therapeutic targets. Recent genomic and transcriptomic analyses have provided valuable insights into EMM; however, a quantitative proteomics analysis using mass spectrometry had not yet been applied for the study of EMM [29,30]. Examining changes at the protein level provides a comprehensive insight into the molecular events underlying EMM development. We used a label-free mass spectrometry approach to effectively quantify the proteomic changes that occur following extramedullary transition. This study has identified 225 SSDA proteins in BMNCs from MM patients with and without EMM. Furthermore, 22 SSDA proteins were detected in blood plasma with three of these proteins being verified as potential biomarkers for the detection of EMM. 

Studies have implicated genetic factors, changes in the bone marrow microenvironment, the differential expression of adhesion molecules and immune evasion in the pathogenesis of EMM [29,30,31,32]. GO and KEGG analysis of proteins increased in abundance in EMM BMNCs revealed an enrichment of cell adhesion associated pathways and biological processes, including the integrin-mediated signalling pathway, cytoskeleton organization, focal adhesion, the Rap1 signalling pathway and, most interestingly, leukocyte transendothelial migration. Eight proteins involved in leukocyte transendothelial migration were increased in abundance in EMM (PECAM1, ITGB1, ACTB, ACTN1, VASP, VCL, RAP1B, RAC1, ROCK2) and may indicate a potential mechanism by which specific MM clones exit the bone marrow niche during extramedullary transition. The dynamic regulation of adhesion proteins during the intravasation of MM cells from the bone marrow has not been fully elucidated. One study reported that the loss of VLA4 (integrin α4 and integrin β1) increases extramedullary disease burden, whereas a recent transcriptomic analysis found that integrin α4 and integrin β1 are co-expressed on EMM cells [30,33]. PECAM1 (CD31) had previously been found to be expressed at higher levels in extramedullary plasmacytomas compared to primary MM cells [34]. 

Several proteins involved in the formation of focal adhesions, which are required to generate mechanical force during migration, were increased in EMM BMNCs [35]. An important component of focal adhesions is integrin linked kinase (ILK), which, when bound to LIM and senescent cell antigen-like-containing domain protein 1 (LIMS1/PINCH1) and β-Parvin, forms the ILK-PINCH-Parvin (IPP) complex [36]. ILK and the IPP complex promote metastasis by promoting a variety of cellular processes, including epithelial mesenchymal transition (EMT) and cell motility [37]. ILK, LIMS1, β-Parvin and another binding partner of LIMS1, Ras suppressor protein 1 (RSU1), were significantly increased in EMM BMNCs (Table S1). A recent study reported that ILK promotes lung adenocarcinoma progression and metastasis through the regulation of KRAS, the IPP complex and RSU1, with other studies also linking ILK to cancer metastasis [38,39,40]. Despite ILK being considered dispensable for myeloma cell survival, inhibiting ILK has previously been shown to reduce the invasive capabilities of myeloma cell lines [41,42]. This combined with the increased abundance of numerous components of the ILK signalling pathway in EMM BMNCs indicates a potential role of this signalling pathway in the migration of myeloma cells to extramedullary sites.

Proteins involved in the tricarboxylic acid (TCA) pathway were decreased in EMM BMNCs compared to MM BMNCs, indicating a potential metabolic change during extramedullary transition. Previous transcriptomic analysis reported the emergence of a metabolic cluster involving pyruvate kinase (PKM2) during extramedullary transition [30]. In this study, PKM2 levels were increased in EMM mononuclear cells. PKM2 has previously been linked to myeloma proliferation and adhesion, reporting that the silencing of PKM2 promoted cell adhesion in cell lines [43]. Interestingly, in lung cancer, secreted PKM2 was found to promote metastasis through interaction with integrin β1, which was also found to be increased in EMM BMNCs [44]. Further validation is required to fully elucidate the role of PKM2 and integrin β1 in the development of EMM.

EMM is frequently associated with an immature or plasmablastic morphology [11,45]. Interestingly, several of the cytoskeletal proteins increased in EMM BMNCs included proteins associated with a plasmablastic morphology (CNN2, PFN1, TMOD3, VASP, TLN1, TMSB4X, PLEK, ZYX) [6]. The hypoxic environment of the bone marrow promotes an immature phenotype in MM through the decreased expression of terminal differentiation markers such as syndecan 1 (CD138) [46]. The endoglycosidase, heparanase, has also been reported to promote myeloma stemness [47]. Our study found decreased levels of CD138 and increased levels of heparanase in the EMM mononuclear fraction. Heparanase promotes an invasive phenotype in MM through the cleavage of CD138 from the surface of MM cells. Shed CD138 subsequently binds to vascular endothelial cell growth factor receptor-2 (VEGFR2) to trigger the polarised migration of MM cells [48]. In EMM, the increased abundance of heparanase may contribute to the creation of a pro-migratory niche within the bone marrow. Further investigations should be performed to elucidate the roles of these proteins associated with the more aggressive, plasmablastic phenotype in extramedullary transition.

Proteomic analysis of EMM plasma identified VCAM1, PEDF and HGFA as potential markers of extramedullary myeloma. VCAM1, a member of the IgG immunoglobulin family, plays a well-known role in cancer development and progression [49]. In MM, the increased expression of VCAM1 and its receptor, VLA-4, correlates with disease progression, and increased levels of soluble VCAM1 (sVCAM1) in MM correlate with advanced disease and poor OS [50,51]. Soluble VCAM1 can be derived from endothelial cells, leukocytes and/or tumour cells via cleavage by metalloproteinases [52]. Interestingly, C-X-C chemokine receptor type 4 (CXCR4), a receptor widely reported to regulate extramedullary myeloma, was found to induce VCAM1 secretion in non-small cell lung cancer via the regulation of the metalloproteinase, ADAM17 [15,53]. The decreased expression of VCAM1 in the bone marrow microenvironment may induce the egress of B cells into circulation, while sVCAM1 has been widely reported to promote lymphocyte migration or stimulate lymphocyte chemotaxis [54,55,56]. The source of the increased concentration of VCAM1 in plasma is unknown and may derive from the shedding of VCAM1 from the surface of MM cells or from other cells known to express VCAM1, such as activated endothelial cells. Nonetheless, sVCAM1 represents a promising marker of EMM and warrants further investigation in a larger cohort of samples. 

The increased levels of sVCAM1 correlated with increased sensitivity to the BCL-2 inhibitors, venetoclax and navitoclax, indicating a potential correlation with BCL-2 expression. VCAM1 and BCL2 are target genes of the nuclear factor kappa B (NF-κB) signalling pathway, a key pathway in MM pathogenesis [57]. Enhanced NF-κB activation may increase sVCAM1 and BCL2 levels, making sVCAM1 a potential surrogate plasma-based biomarker of response to BCL-2 inhibitors; however, further studies in a larger cohort of patients are required to further evaluate this link. The extrinsic activation of the NF-κB pathway via APRIL and BAFF provides survival signals in the early stages of MM, whereas during tumour progression, mutations in NF-κB pathway genes can result in autonomous NF-κB pathway activation and reduced dependence on the bone marrow microenvironment [58]. The link between EMM and the constitutive activation of the NF-κB has yet to be evaluated.

The monomeric glycoprotein, PEDF, was significantly increased in EMM plasma compared to the plasma of MM patients without extramedullary spread. PEDF has been widely reported as an anti-angiogenic and anti-tumorigenic protein in many cancers [59]. Literature focusing on PEDF in MM found that PEDF suppresses VEGF signalling and inhibits multiple myeloma through the inhibition of reactive oxygen species (ROS) generation [60,61]. In contrast, increased PEDF has been implicated in promoting metastasis and invasion in several cancers including hepatocellular carcinoma and oesophageal squamous cell carcinoma [62,63]. A recent study provides insight into the conflicting reports on PEDF as a metastatic biomarker and suggested a potential dual role depending on tissue type and stage of metastasis [64]. Therefore, an in-depth, focused investigation may determine the specific role of PEDF in the extramedullary transition of MM [65].

HGFA is a serine protease that catalyses the activation hepatocyte growth factor (HGF). MM cell lines and primary myeloma cells secrete HGFA which can then activate HGF [66]. Furthermore, HGFA has been reported to be present in high levels in the sera and bone marrow of MM patients [67]. HGF is a pleiotropic cytokine involved in the progression of the monoclonal gammopathy of undetermined significance (MGUS) to MM, myeloma cell proliferation and survival [68]. Interestingly, heparanase increases the expression and secretion of HGF in MM, and secreted HGF can form an active complex with cleaved CD138 which promotes c-Met signalling [69]. Combining HGFA, VCAM1 and PEDF into a three-marker panel for the detection of EMM using logistic regression analysis increased the discriminatory power when compared to the individual proteins. Further investigation on the use of this panel to detect EMM at an early stage is needed. The mechanisms driving the increase in circulating VCAM1, HGFA and PEDF in EMM plasma are currently unknown. Future mechanistic studies involving these proteins will provide more insight into the cellular processes that lead to increased levels of VCAM1, HGFA and PEDF in EMM plasma.

Targeted metabolomics/lipidomics analysis identified 26 lipids, tyrosine and GUDCA as increased in abundance in EMM plasma, and taurine, phenylalanine betaine and PC aa C38:1 as decreased in EMM plasma, indicating a distinct plasma metabolite profile in EMM compared to medullary MM. A trend towards an increase in triglyceride concentrations in the plasma of patients with EMM was also identified. Several EMM case studies have reported hyperlipidaemia, which is more predominant in patients with IgA myeloma [70,71,72]. MM patients with the IgA monoclonal protein type are also at higher risk of future EMM development [73]. Several studies have analysed triglyceride levels in healthy controls and MM patients, finding no change in triglyceride levels apart from one study which noted an increase in triglycerides during the active disease period [74]. The increased lipid levels observed in EMM plasma may indicate a link between dysregulated lipid metabolism and EMM. A recent study found that targeting fatty acid binding proteins (FABPs), including FABP5, in MM reduced MYC signalling and induced apoptosis of myeloma cells, highlighting the association of aberrant lipid metabolism with MM [75]. The results of the cellular GO analysis in this study indicate a clear metabolic change, emphasised by the reduced abundance of TCA cycle proteins in EMM BMNCs. CD36, a fatty acid transporter that enhances fatty acid uptake into cells similarly to FABPs, was increased in abundance in EMM BMNCs and may contribute to abnormal lipid metabolism within the BME [76]. Statins are commonly used to lower lipid concentrations by targeting the mevalonate pathway. Statin use has been reported to reduce the risk of MM and improve MM survival rates, although the biological mechanisms have not been fully elucidated [77,78]. Recent evidence demonstrate that statins may act as metastasis inhibitors in various solid cancers, including colon cancer [79,80]. Statins may represent a promising approach to target lipid metabolism in EMM; however, further investigation is required to evaluate this. A strong anti-tumorigenic role of taurine has been reported; however, this has not been thoroughly analysed in MM [56].

The efficacy of current therapeutics in the treatment of EMM is limited, as exemplified by the known poor prognosis of patients who present with extramedullary lesions [81,82]. Despite developments in the treatment of MM through the introduction of immunotherapies, preliminary studies indicate that the long-term efficacy of these treatments is significantly worse in patients with EMM compared to MM patients without extramedullary spread [83,84]. Our study illustrates a clear phenotypic change in the bone marrow niche of EMM patients compared to MM patients without extramedullary spread, suggesting a need for novel drug combinations or drug targets for the treatment of EMM to improve patient prognosis and treatment response. The bone marrow microenvironment can influence the dissemination of myeloma cells. Targeting myeloma clones with capacity for extramedullary spread in the context of the bone marrow microenvironment may be a promising approach to limit extramedullary transition [21,85]. As increased levels of sVCAM1 were associated with EMM and correlated with sensitivity to BCL2 inhibitors, venetoclax and navitoclax may represent promising therapeutics for the treatment of EMM. Several proteins found to be increased in the bone marrow mononuclear fraction of EMM patients, such as heparanase and ROCK2, have specific inhibitors available that warrant investigation in the context of EMM progression (Table 5) [86,87]. Crucially, large multi-centre studies are required to incorporate satisfactory sample sizes to comprehensively evaluate the molecular mechanisms associated with EMM and the efficacy of novel drug combinations in EMM.

Our paper includes a small sample size and lacks cellular proteomic verification. This is due to the fact that EMM is a rare manifestation of multiple myeloma which limits the availability of clinical samples for initial analysis and subsequent validation. The use of the MMRF CoMMpass dataset to determine the prognostic value of the most significantly increased proteins in EMM BMNCs provides some insight into the association of these proteins with more aggressive disease; however, validation in an independent cohort of EMM patients would improve our confidence in the association of these proteins with EMM transition. Finally, BMNCs from EMM and MM patients were used for proteomic analysis, which means that proteomic changes seen between the two groups are not solely associated with myeloma cells and instead associated with changes in the mononuclear fraction. However, with the growing use of monoclonal and bispecific antibodies in the treatment of MM, analysing various cells from the bone marrow microenvironment is relevant.

Within these limitations, however, this study shows that the proteomic alterations in the bone marrow and plasma of patients with and without EMM is impactful. We assumed that the presence of extramedullary lesions is derived from changes in the bone marrow microenvironment, and we evaluated the change in the proteomic profile of the bone marrow and plasma in the context of EMM. The potential plasma biomarkers we identified may represent factors produced by myeloma cells from extramedullary lesions. Further molecular analyses and larger scaled studies are needed to explore and confirm the link between the proteins identified in this study and EMM more definitively.

## 5. Conclusions

To the best of our knowledge, this pilot study using label-free mass spectrometry to evaluate proteomic changes in MM patients with and without extramedullary spread is the first of its kind. Determining the underlying molecular processes involved in the development of EMM is crucial to advancing patient care. VCAM1, PEDF and HGFA warrant further investigation as markers of extramedullary transition in a larger cohort of patients. Ultimately, this study illustrates that extramedullary myeloma is phenotypically different to medullary myeloma and, as such, warrants a different therapeutic approach with novel drug targets and drug combinations to improve survival rates. We hope this proteomic study will inform future experimental designs and research in EMM. 

## Figures and Tables

**Table 1:** Clinical and demographic characteristics of patients involved in this study. Characteristics include diagnosis, status at diagnosis, sex, age and overall survival.

Sample ID	Diagnosis	Status	Sex	Age	OS (mo) from Diagnosis
D_EMM_2689	Myeloma, extramedullary	Diagnostic	Male	65	80
D_EMM_3497	Myeloma, extramedullary	Diagnostic	Male	65	87 *
D_EMM_3674	Myeloma, extramedullary	Diagnostic	Male	58	8
D_EMM_4296	Myeloma, extramedullary	Diagnostic	Male	65	22
D_EMM_1994	Myeloma, extramedullary	Diagnostic	Female	67	16
D_EMM_40725	Myeloma, extramedullary	Diagnostic	Male	49	2
PD_EMM_874	Myeloma, extramedullary	Progressive disease	Male	72	31
PD_EMM_1994 ^†^	Myeloma, extramedullary	Progressive disease	Female	68	16
PD_EMM_40795	Myeloma, extramedullary	Progressive disease	Female	69	7
D_MM_5215	Myeloma, no extramedullary	Diagnostic	Male	65	61 *
D_MM_4314	Myeloma, no extramedullary	Diagnostic	Male	65	53
D_MM_5187	Myeloma, no extramedullary	Diagnostic	Male	59	62 *
D_MM_4317	Myeloma, no extramedullary	Diagnostic	Male	65	65
D_MM_40141	Myeloma, no extramedullary	Diagnostic	Male	49	43 *
PD_MM_899	Myeloma, no extramedullary	Progressive disease	Male	72	124
PD_MM_1579	Myeloma, no extramedullary	Progressive disease	Female	68	83
PD_MM_40301	Myeloma, no extramedullary	Progressive disease	Female	70	129 *

* Patient was alive at last follow-up. ^†^ D_EMM_1994 and PD_EMM_1994 were collected from the same patient. PD_EMM_1994 sample was collected approximately 1 year after D_EMM_1994.

**Table 2:** Top 25 proteins with significantly increased abundance in EMM BMNCs compared to MM BMNCs as determined by label-free LC-MS/MS.

Uniprot ID	Description	Gene Name	Fold Change	FDR-Adjusted *p*-Value
P00918	Carbonic anhydrase 2	CA2	4.42	0.0001
Q8NBJ5	Procollagen galactosyltransferase 1	COLGALT1	1.77	0.0003
P09382	Galectin-1	LGALS1	1.94	0.0005
Q5JRX3	Presequence protease, mitochondrial	PITRM1	3.05	0.0006
P37802	Transgelin-2	TAGLN2	4.65	0.0006
P17301	Integrin alpha-2	ITGA2	34.90	0.0009
Q86WV6	Stimulator of interferon genes protein	TMEM173	3.02	0.0009
Q32MZ4	Leucine-rich repeat flightless-interacting protein 1	LRRFIP1	2.13	0.0009
Q15833	Syntaxin-binding protein 2	STXBP2	2.26	0.0011
P62328	Thymosin beta-4	TMSB4X	8.64	0.0011
P08567	Pleckstrin	PLEK	5.45	0.0015
Q9UGT4	Sushi domain-containing protein 2	SUSD2	53.35	0.0017
O60610	Protein diaphanous homolog 1	DIAPH1	2.21	0.0018
P08758	Annexin A5	ANXA5	7.68	0.0018
P07951	Tropomyosin beta chain	TPM2	3.45	0.0019
Q7LDG7	RAS guanyl-releasing protein 2	RASGRP2	3.54	0.0019
Q14019	Coactosin-like protein	COTL1	2.38	0.0020
P18054	Polyunsaturated fatty acid lipoxygenase ALOX12	ALOX12	26.46	0.0020
Q9NYL9	Tropomodulin-3	TMOD3	2.90	0.0020
P63000	Ras-related C3 botulinum toxin substrate 1	RAC1	2.73	0.0025
P37840	Alpha-synuclein	SNCA	18.57	0.0025
Q9HBI1	Beta-parvin	PARVB	12.09	0.0026
P18206	Vinculin	VCL	5.78	0.0028
Q15942	Zyxin	ZYX	4.93	0.0029
P06753	Tropomyosin alpha-3 chain	TPM3	2.07	0.0030

**Table 3:** Top 25 proteins with significantly decreased abundance in EMM BMNCs compared to MM BMNCs as determined by label-free LC-MS/MS.

Uniprot ID	Description	Gene Name	Fold Change	FDR Adjusted *p*-Value
P22087	rRNA 2′-O-methyltransferase fibrillarin	FBL	1.65	0.0003
P16402	Histone H1.3	HIST1H1D	2.89	0.0007
Q8NBS9	Thioredoxin domain-containing protein 5	TXNDC5	4.33	0.0008
Q99798	Aconitate hydratase, mitochondrial	ACO2	1.61	0.0012
Q9NSE4	Isoleucine--tRNA ligase, mitochondrial	IARS2	2.22	0.0014
Q9Y320	Thioredoxin-related transmembrane protein 2	TMX2	5.92	0.0014
Q13263	Transcription intermediary factor 1-beta	TRIM28	2.19	0.0015
P30837	Aldehyde dehydrogenase X, mitochondrial	ALDH1B1	8.19	0.0015
Q9BY50	Signal peptidase complex catalytic subunit SEC11C	SEC11C	5.19	0.0016
Q13813	Spectrin alpha chain, non-erythrocytic 1	SPTAN1	2.10	0.0023
Q3SY69	Mitochondrial 10-formyltetrahydrofolate dehydrogenase	ALDH1L2	7.33	0.0033
P08240	Signal recognition particle receptor subunit alpha	SRPR	2.48	0.0035
P30044	Peroxiredoxin-5, mitochondrial	PRDX5	1.77	0.0037
Q7KZF4	Staphylococcal nuclease domain-containing protein 1	SND1	2.91	0.0042
P49257	Protein ERGIC-53	LMAN1	2.44	0.0043
Q9Y4P3	Transducin beta-like protein 2	TBL2	4.53	0.0045
P09874	Poly [ADP-ribose] polymerase 1	PARP1	2.67	0.0047
Q01105	Protein SET	SET	3.29	0.0054
Q92506	Estradiol 17-beta-dehydrogenase 8	HSD17B8	3.37	0.0054
P12235	ADP/ATP translocase 1	SLC25A4	4.43	0.0055
Q13310	Polyadenylate-binding protein 4	PABPC4	4.47	0.0056
P53992	Protein transport protein Sec24C	SEC24C	38.68	0.0057
Q16706	Alpha-mannosidase 2	MAN2A1	6.43	0.0058
Q01082	Spectrin beta chain, non-erythrocytic 1	SPTBN1	1.96	0.0060
P54886	Delta-1-pyrroline-5-carboxylate synthase	ALDH18A1	10.26	0.0072

**Table 4:** List of proteins significantly increased in abundance in the plasma of EMM patients compared to MM patients without extramedullary spread.

Uniprot ID	Description	Gene Name	Fold Change	FDR-Adjusted *p*-Value
Q9NZP8	Complement C1r subcomponent-like protein	C1RL	1.58	0.012
P02747	Complement C1q subcomponent subunit C	C1QC	2.45	0.030
P36955	Pigment epithelium-derived factor	SERPINF1	1.56	0.031
P23470	Receptor-type tyrosine-protein phosphatase gamma	PTPRG	2.87	0.035
P02745	Complement C1q subcomponent subunit A	C1QA	2.33	0.038
Q04756	Hepatocyte growth factor activator	HGFA	2.09	0.038
P05062	Fructose-bisphosphate aldolase B	ALDOB	2.31	0.039
P02746	Complement C1q subcomponent subunit B	C1QB	2.66	0.041
P22891	Vitamin K-dependent protein Z	PROZ	1.83	0.042
P06276	Cholinesterase	BCHE	1.88	0.056
Q9HDC9	Adipocyte plasma membrane-associated protein	APMAP	1.85	0.058
P02760	Protein AMBP	AMBP	1.69	0.062
P07359	Platelet glycoprotein Ib alpha chain	GP1BA	2.40	0.070
P19320	Vascular cell adhesion protein 1	VCAM1	2.23	0.078
P01023	Alpha-2-macroglobulin	A2M	2.50	0.079
P00736	Complement C1r subcomponent	C1R	1.63	0.079
P15144	Aminopeptidase N	ANPEP	2.65	0.081
P01871	Ig mu chain C region	IGHM	4.84	0.083
P04114	Apolipoprotein B-100	APOB	1.54	0.083
Q86UD1	Out at first protein homolog	OAF	2.24	0.085
P0C0L5	Complement C4-B	C4B	1.88	0.085
Q12913	Receptor-type tyrosine-protein phosphatase eta	PTPRJ	1.79	0.095

**Table 5:** Potential targets/markers and associated therapeutics for the treatment of EMM patients based on the current literature. This table provides a rationale for future studies focusing on the detection of drug targets in EMM. BCL2, B-cell lymphoma 2; qPCR, quantitative polymerase chain reaction; BCL-XL, B-cell lymphoma—extra large; XPO1, exportin 1; MEK, mitogen-activated protein kinase kinase; BRAF, B-Raf.

Protein Target/Marker	Potential Therapeutic	Method of Target Detection	FDA Approval	References
Potential protein targets in extramedullary multiple myeloma (identified from the literature)				
BCL2	Venetoclax	Immunohistochemistry, qPCR, fl–w cytometry	Yes—Acute myeloid leukaemia, Chronic lymphocytic leukaemia	[88,89]
BCL2, BCL-XL	Navitoclax	Immunohistochemistry, qPCR, flow cytometry	No	[89,90]
XPO1	Selinexor	Immunohistochemistry	Yes—Multiple myeloma	[91,92]
Aminopeptidase expression (Correlates with Melflufen sensitivity)	Melflufen	RNA sequencing	No	[93,94]
MEK	Trametinib	Targeted sequencing for RAS mutations	Yes (in combination with dabrafenib)–Various metastatic solid tumours with BRAF V600 E mutation	[11,95]
CD44v	4SCAR-CD44v6	Immunohistochemistry, flow cytometry	No	[96,97]
BRAF V600E	Vemurafenib, encorafenib, binimetinib	Allele-specific PCR	Yes–Metastatic melanoma with BRAF V600 E mutation	[98,99]
Potential protein targets in extramedullary multiple myeloma (identified in this study)				
LGALS1	OTX008	Immunohistochemistry	No	[100,101,102]
HPSE	Roneparstat	Immunohistochemistry	No	[86]
ROCK2	Belumosudil	qPCR, immunohistochemistry	Yes–Chronic graft-versus-host disease	[103]
ILK	QLT0267, Compound 22	Immunohistochemistry	No	[104,105]
Lipids	Statins	Unknown	Yes	[78,79]

## Data Availability

The data presented in this study are available on request from the corresponding author. The data are not publicly available due to privacy and ethical limitations.

## Supplementary Materials

The following supporting information can be downloaded at: https://www.mdpi.com/article/10.3390/cancers15153764/s1: Figure S1: Cytogenetics of patient cohort; Figure S2: Survival graph illustrating the difference in OS between the EMM group (*n* = 8) and medullary MM group (*n* = 8); Figure S3: Survival graphs illustrating the difference in OS between patients with high expression and low expression of the seven proteins identified as potential prognostic biomarkers in the CoMMpass dataset. Samples were divided based on median expression levels. (A) TAGLN2, (B) CA2, (C) ITGA2, (D) LGALS1, (E) TPM2, (F) TMOD3, (G) TPM3; Figure S4: Targeted metabolomic analysis shows a trend towards increased triglycerides and lipids in the plasma of EMM patients (A) Total plasma triglyceride concentration in MM patients with and without extramedullary spread. (B) Total lipid concentration in MM patients with and without extramedullary spread; Figure S5: Spearman’s correlation matrix between differential metabolites and proteins in plasma. Table S1: Full list of statistically significant differentially abundant (SSDA) proteins in EMM bone marrow mononuclear cells (BMNCs) and MM BMNCs; Table S2: Metabolites with significant differential abundance in the plasma of MM and EMM patients.

## References

[1] Kumar S.K., Rajkumar V., Kyle R.A., van Duin M., Sonneveld P., Mateos M.-V., Gay F., Anderson K.C. (2017). Multiple Myeloma. Nat. Rev. Dis. Primers.

[2] Pinto V., Bergantim R., Caires H.R., Seca H., Guimarães J.E., Vasconcelos M.H. (2020). Multiple Myeloma: Available Therapies and Causes of Drug Resistance. Cancers.

[3] van de Donk N.W.C.J., Pawlyn C., Yong K.L. (2021). Multiple Myeloma. Lancet.

[4] Rajkumar S.V. (2016). Updated Diagnostic Criteria and Staging System for Multiple Myeloma. Am. Soc. Clin. Oncol. Educ. Book.

[5] Bhutani M., Foureau D.M., Atrash S., Voorhees P.M., Usmani S.Z. (2020). Extramedullary Multiple Myeloma. Leukemia.

[6] Gagelmann N., Eikema D.-J., Iacobelli S., Koster L., Nahi H., Stoppa A.-M., Masszi T., Caillot D., Lenhoff S., Udvardy M. (2018). Impact of Extramedullary Disease in Patients with Newly Diagnosed Multiple Myeloma Undergoing Autologous Stem Cell Transplantation: A Study from the Chronic Malignancies Working Party of the EBMT. Haematologica.

[7] Beksac M., Seval G.C., Kanellias N., Coriu D., Rosiñol L., Ozet G., Goranova-Marinova V., Unal A., Bila J., Ozsan H. (2020). A Real World Multicenter Retrospective Study on Extramedullary Disease from Balkan Myeloma Study Group and Barcelona University: Analysis of Parameters That Improve Outcome. Haematologica.

[8] Bladé J., Beksac M., Caers J., Jurczyszyn A., von Lilienfeld-Toal M., Moreau P., Rasche L., Rosiñol L., Usmani S.Z., Zamagni E. (2022). Extramedullary Disease in Multiple Myeloma: A Systematic Literature Review. Blood Cancer J..

[9] Moreau P., Attal M., Caillot D., Macro M., Karlin L., Garderet L., Facon T., Benboubker L., Escoffre-Barbe M., Stoppa A.-M. (2017). Prospective Evaluation of Magnetic Resonance Imaging and [18F]Fluorodeoxyglucose Positron Emission Tomography-Computed Tomography at Diagnosis and Before Maintenance Therapy in Symptomatic Patients with Multiple Myeloma Included in the IFM/DFCI 2009 Trial: Results of the IMAJEM Study. J. Clin. Oncol..

[10] Badar T., Srour S., Bashir Q., Shah N., Al-Atrash G., Hosing C., Popat U., Nieto Y., Orlowski R.Z., Champlin R. (2017). Predictors of Inferior Clinical Outcome in Patients with Standard-Risk Multiple Myeloma. Eur. J. Haematol..

[11] Touzeau C., Moreau P. (2016). How I Treat Extramedullary Myeloma. Blood.

[12] Billecke L., Murga Penas E.M., May A.M., Engelhardt M., Nagler A., Leiba M., Schiby G., Kröger N., Zustin J., Marx A. (2013). Cytogenetics of Extramedullary Manifestations in Multiple Myeloma. Br. J. Haematol..

[13] García-Ortiz A., Rodríguez-García Y., Encinas J., Maroto-Martín E., Castellano E., Teixidó J., Martínez-López J. (2021). The Role of Tumor Microenvironment in Multiple Myeloma Development and Progression. Cancers.

[14] Solimando A., Vià M.D., Croci G., Borrelli P., Tabares P., Brandl A., Munawar U., Steinbrunn T., Balduini A., Rauert-Wunderlich H. (2021). OAB-041: Epithelial-Mesenchymal-Transition Regulated by Junctional Adhesion Molecule-A (JAM-A) Associates with Aggressive Extramedullary Multiple Myeloma Disease. Clin. Lymphoma Myeloma Leuk..

[15] Roccaro A.M., Mishima Y., Sacco A., Moschetta M., Tai Y.-T., Shi J., Zhang Y., Reagan M.R., Huynh D., Kawano Y. (2015). CXCR4 Regulates Extra-Medullary Myeloma through Epithelial-Mesenchymal-Transition-like Transcriptional Activation. Cell Rep..

[16] Greipp P.R., Leong T., Bennett J.M., Gaillard J.P., Klein B., Stewart J.A., Oken M.M., Kay N.E., Van Ness B., Kyle R.A. (1998). Plasmablastic Morphology—An Independent Prognostic Factor with Clinical and Laboratory Correlates: Eastern Cooperative Oncology Group (ECOG) Myeloma Trial E9486 Report by the ECOG Myeloma Laboratory Group. Blood.

[17] Liu Y., Jelloul F., Zhang Y., Bhavsar T., Ho C., Rao M., Lewis N.E., Cimera R., Baik J., Sigler A. (2020). Genetic Basis of Extramedullary Plasmablastic Transformation of Multiple Myeloma. Am. J. Surg. Pathol..

[18] Weinstock M., Ghobrial I.M. (2013). Extramedullary Multiple Myeloma. Leuk. Lymphoma.

[19] Wiśniewski J.R., Zougman A., Nagaraj N., Mann M. (2009). Universal Sample Preparation Method for Proteome Analysis. Nat. Methods.

[20] Raudvere U., Kolberg L., Kuzmin I., Arak T., Adler P., Peterson H., Vilo J. (2019). G:Profiler: A Web Server for Functional Enrichment Analysis and Conversions of Gene Lists (2019 Update). Nucleic Acids Res..

[21] Forster S., Radpour R. (2022). Molecular Impact of the Tumor Microenvironment on Multiple Myeloma Dissemination and Extramedullary Disease. Front. Oncol..

[22] Majumder M.M., Silvennoinen R., Anttila P., Tamborero D., Eldfors S., Yadav B., Karjalainen R., Kuusanmäki H., Lievonen J., Parsons A. (2017). Identification of Precision Treatment Strategies for Relapsed/Refractory Multiple Myeloma by Functional Drug Sensitivity Testing. Oncotarget.

[23] Tierney C., Bazou D., Majumder M.M., Anttila P., Silvennoinen R., Heckman C.A., Dowling P., O’Gorman P. (2021). Next Generation Proteomics with Drug Sensitivity Screening Identifies Sub-Clones Informing Therapeutic and Drug Development Strategies for Multiple Myeloma Patients. Sci. Rep..

[24] Godzien J., Ciborowski M., Angulo S., Barbas C. (2013). From Numbers to a Biological Sense: How the Strategy Chosen for Metabolomics Data Treatment May Affect Final Results. A Practical Example Based on Urine Fingerprints Obtained by LC-MS. Electrophoresis.

[25] An R., Yu H., Wang Y., Lu J., Gao Y., Xie X., Zhang J. (2022). Integrative Analysis of Plasma Metabolomics and Proteomics Reveals the Metabolic Landscape of Breast Cancer. Cancer Metab..

[26] Sevcikova S., Minarik J., Stork M., Jelinek T., Pour L., Hajek R. (2019). Extramedullary Disease in Multiple Myeloma—Controversies and Future Directions. Blood Rev..

[27] Ormond Filho A.G., Carneiro B.C., Pastore D., Silva I.P., Yamashita S.R., Consolo F.D., Hungria V.T.M., Sandes A.F., Rizzatti E.G., Nico M.A.C. (2019). Whole-Body Imaging of Multiple Myeloma: Diagnostic Criteria. RadioGraphics.

[28] Gozzetti A., Kok C.H., Li C.-F. (2022). Editorial: Molecular Mechanisms of Multiple Myeloma. Front. Oncol..

[29] Kriegova E., Fillerova R., Minarik J., Savara J., Manakova J., Petrackova A., Dihel M., Balcarkova J., Krhovska P., Pika T. (2021). Whole-Genome Optical Mapping of Bone-Marrow Myeloma Cells Reveals Association of Extramedullary Multiple Myeloma with Chromosome 1 Abnormalities. Sci. Rep..

[30] Ryu D., Kim S.J., Hong Y., Jo A., Kim N., Kim H.-J., Lee H.-O., Kim K., Park W.-Y. (2020). Alterations in the Transcriptional Programs of Myeloma Cells and the Microenvironment during Extramedullary Progression Affect Proliferation and Immune Evasion. Clin. Cancer Res..

[31] Gregorova J., Vychytilova-Faltejskova P., Kramarova T., Knechtova Z., Almasi M., Stork M., Pour L., Kohoutek J., Sevcikova S. (2022). Proteomic Analysis of the Bone Marrow Microenvironment in Extramedullary Multiple Myeloma Patients. Neoplasma.

[32] Bou Zerdan M., Nasr L., Kassab J., Saba L., Ghossein M., Yaghi M., Dominguez B., Chaulagain C.P. (2022). Adhesion Molecules in Multiple Myeloma Oncogenesis and Targeted Therapy. Int. J. Hematol. Oncol..

[33] Hathi D., Chanswangphuwana C., Cho N., Fontana F., Maji D., Ritchey J., O’Neal J., Ghai A., Duncan K., Akers W.J. (2022). Ablation of VLA4 in Multiple Myeloma Cells Redirects Tumor Spread and Prolongs Survival. Sci. Rep..

[34] Hedvat C.V., Comenzo R.L., Teruya-Feldstein J., Olshen A.B., Ely S.A., Osman K., Zhang Y., Kalakonda N., Nimer S.D. (2003). Insights into Extramedullary Tumour Cell Growth Revealed by Expression Profiling of Human Plasmacytomas and Multiple Myeloma. Br. J. Haematol..

[35] Nagano M., Hoshino D., Koshikawa N., Akizawa T., Seiki M. (2012). Turnover of Focal Adhesions and Cancer Cell Migration. Int. J. Cell Biol..

[36] Górska A., Mazur A.J. (2022). Integrin-Linked Kinase (ILK): The Known vs. the Unknown and Perspectives. Cell. Mol. Life Sci..

[37] McDonald P.C., Dedhar S. (2022). New Perspectives on the Role of Integrin-Linked Kinase (ILK) Signaling in Cancer Metastasis. Cancers.

[38] Nikou S., Arbi M., Dimitrakopoulos F.-I.D., Sirinian C., Chadla P., Pappa I., Ntaliarda G., Stathopoulos G.T., Papadaki H., Zolota V. (2020). Integrin-Linked Kinase (ILK) Regulates KRAS, IPP Complex and Ras Suppressor-1 (RSU1) Promoting Lung Adenocarcinoma Progression and Poor Survival. J. Mol. Hist..

[39] Tsoumas D., Nikou S., Giannopoulou E., Tsaniras S.C., Sirinian C., Maroulis I., Taraviras S., Zolota V., Kalofonos H.P., Bravou V. (2018). ILK Expression in Colorectal Cancer Is Associated with EMT, Cancer Stem Cell Markers and Chemoresistance. Cancer Genom. Proteom..

[40] Chen D., Zhang Y., Zhang X., Li J., Han B., Liu S., Wang L., Ling Y., Mao S., Wang X. (2013). Overexpression of Integrin-Linked Kinase Correlates with Malignant Phenotype in Non-Small Cell Lung Cancer and Promotes Lung Cancer Cell Invasion and Migration via Regulating Epithelial–Mesenchymal Transition (EMT)-Related Genes. Acta Histochem..

[41] Wang X., Zhang Z., Yao C. (2011). Targeting Integrin-Linked Kinase Increases Apoptosis and Decreases Invasion of Myeloma Cell Lines and Inhibits IL-6 and VEGF Secretion from BMSCs. Med. Oncol..

[42] Steinbrunn T., Siegmund D., Andrulis M., Grella E., Kortüm M., Einsele H., Wajant H., Bargou R.C., Stühmer T. (2012). Integrin-Linked Kinase Is Dispensable for Multiple Myeloma Cell Survival. Leuk. Res..

[43] He Y., Wang Y., Liu H., Xu X., He S., Tang J., Huang Y., Miao X., Wu Y., Wang Q. (2015). Pyruvate Kinase Isoform M2 (PKM2) Participates in Multiple Myeloma Cell Proliferation, Adhesion and Chemoresistance. Leuk. Res..

[44] Wang C., Zhang S., Liu J., Tian Y., Ma B., Xu S., Fu Y., Luo Y. (2020). Secreted Pyruvate Kinase M2 Promotes Lung Cancer Metastasis through Activating the Integrin Beta1/FAK Signaling Pathway. Cell Rep..

[45] Dah K., Lavezo J.L., Dihowm F. (2021). Aggressive Plasmablastic Myeloma with Extramedullary Cord Compression and Hyperammonemic Encephalopathy: Case Report and Literature Review. Anticancer Res..

[46] Muz B., de la Puente P., Azab F., Luderer M., Azab A.K. (2014). Hypoxia Promotes Stem Cell-like Phenotype in Multiple Myeloma Cells. Blood Cancer J..

[47] Tripathi K., Ramani V.C., Bandari S.K., Amin R., Brown E.E., Ritchie J.P., Stewart M.D., Sanderson R.D. (2020). Heparanase Promotes Myeloma Stemness and in Vivo Tumorigenesis. Matrix Biol..

[48] Jung O., Trapp-Stamborski V., Purushothaman A., Jin H., Wang H., Sanderson R.D., Rapraeger A.C. (2016). Heparanase-Induced Shedding of Syndecan-1/CD138 in Myeloma and Endothelial Cells Activates VEGFR2 and an Invasive Phenotype: Prevention by Novel Synstatins. Oncogenesis.

[49] Zhang D., Bi J., Liang Q., Wang S., Zhang L., Han F., Li S., Qiu B., Fan X., Chen W. (2020). VCAM1 Promotes Tumor Cell Invasion and Metastasis by Inducing EMT and Transendothelial Migration in Colorectal Cancer. Front. Oncol..

[50] Hao P., Zhang C., Wang R., Yan P., Peng R. (2020). Expression and Pathogenesis of VCAM-1 and VLA-4 Cytokines in Multiple Myeloma. Saudi J. Biol. Sci..

[51] Terpos E., Migkou M., Christoulas D., Gavriatopoulou M., Eleutherakis-Papaiakovou E., Kanellias N., Iakovaki M., Panagiotidis I., Ziogas D.C., Fotiou D. (2016). Increased Circulating VCAM-1 Correlates with Advanced Disease and Poor Survival in Patients with Multiple Myeloma: Reduction by Post-Bortezomib and Lenalidomide Treatment. Blood Cancer J..

[52] Okugawa Y., Miki C., Toiyama Y., Koike Y., Yokoe T., Saigusa S., Tanaka K., Inoue Y., Kusunoki M. (2010). Soluble VCAM-1 and Its Relation to Disease Progression in Colorectal Carcinoma. Exp. Ther. Med..

[53] Liao T., Chen W., Sun J., Zhang Y., Hu X., Yang S., Qiu H., Li S., Chu T. (2018). CXCR4 Accelerates Osteoclastogenesis Induced by Non-Small Cell Lung Carcinoma Cells Through Self-Potentiation and VCAM1 Secretion. CPB.

[54] Alexiou D., Karayiannakis A.J., Syrigos K.N., Zbar A., Kremmyda A., Bramis I., Tsigris C. (2001). Serum Levels of E-Selectin, ICAM-1 and VCAM-1 in Colorectal Cancer Patients: Correlations with Clinicopathological Features, Patient Survival and Tumour Surgery. Eur. J. Cancer.

[55] Ding Y.-B., Chen G.-Y., Xia J.-G., Zang X.-W., Yang H.-Y., Yang L. (2003). Association of VCAM-1 Overexpression with Oncogenesis, Tumor Angiogenesis and Metastasis of Gastric Carcinoma. World J. Gastroenterol..

[56] Kitani A., Nakashima N., Izumihara T., Inagaki M., Baoui X., Yu S., Matsuda T., Matsuyama T. (1998). Soluble VCAM-1 Induces Chemotaxis of Jurkat and Synovial Fluid T Cells Bearing High Affinity Very Late Antigen-4. J. Immunol..

[57] Roy P., Sarkar U.A., Basak S. (2018). The NF-ΚB Activating Pathways in Multiple Myeloma. Biomedicines.

[58] Demchenko Y.N., Glebov O.K., Zingone A., Keats J.J., Bergsagel P.L., Kuehl W.M. (2010). Classical and/or Alternative NF-ΚB Pathway Activation in Multiple Myeloma. Blood.

[59] Becerra S.P., Notario V. (2013). The Effects of PEDF on Cancer Biology: Mechanisms of Action and Therapeutic Potential. Nat. Rev. Cancer.

[60] Seki R., Yoshida T., Nakamura K., Yamagishi S., Imaizumi T., Okamura T., Sata M. (2005). Pigment Epithelium-Derived Factor (PEDF) Inhibits Multiple Myeloma through Suppressing NADPH Oxidase ROS Generation. Blood.

[61] Seki R., Yamagishi S., Matsui T., Yoshida T., Torimura T., Ueno T., Sata M., Okamura T. (2013). Pigment Epithelium-Derived Factor (PEDF) Inhibits Survival and Proliferation of VEGF-Exposed Multiple Myeloma Cells through Its Anti-Oxidative Properties. Biochem. Biophys. Res. Commun..

[62] Chen Z., Che D., Gu X., Lin J., Deng J., Jiang P., Xu K., Xu B., Zhang T. (2021). Upregulation of PEDF Predicts a Poor Prognosis and Promotes Esophageal Squamous Cell Carcinoma Progression by Modulating the MAPK/ERK Signaling Pathway. Front. Oncol..

[63] Hou J., Ge C., Cui M., Liu T., Liu X., Tian H., Zhao F., Chen T., Cui Y., Yao M. (2017). Pigment Epithelium-Derived Factor Promotes Tumor Metastasis through an Interaction with Laminin Receptor in Hepatocellular Carcinomas. Cell Death Dis..

[64] Abooshahab R., Al-Salami H., Dass C.R. (2021). The Increasing Role of Pigment Epithelium-Derived Factor in Metastasis: From Biological Importance to a Promising Target. Biochem. Pharmacol..

[65] Kuriyama S., Tanaka G., Takagane K., Itoh G., Tanaka M. (2022). Pigment Epithelium Derived Factor Is Involved in the Late Phase of Osteosarcoma Metastasis by Increasing Extravasation and Cell-Cell Adhesion. Front. Oncol..

[66] Tjin E.P.M., Derksen P.W.B., Kataoka H., Spaargaren M., Pals S.T. (2004). Multiple Myeloma Cells Catalyze Hepatocyte Growth Factor (HGF) Activation by Secreting the Serine Protease HGF-Activator. Blood.

[67] Wader K.F., Fagerli U.M., Holt R.U., Stordal B., Børset M., Sundan A., Waage A. (2008). Elevated Serum Concentrations of Activated Hepatocyte Growth Factor Activator in Patients with Multiple Myeloma. Eur. J. Haematol..

[68] Giannoni P., de Totero D. (2021). The HGF/c-MET Axis as a Potential Target to Overcome Survival Signals and Improve Therapeutic Efficacy in Multiple Myeloma. Cancer Drug Resist..

[69] Ramani V.C., Yang Y., Ren Y., Nan L., Sanderson R.D. (2011). Heparanase Plays a Dual Role in Driving Hepatocyte Growth Factor (HGF) Signaling by Enhancing HGF Expression and Activity. J. Biol. Chem..

[70] Misselwitz B., Goede J.S., Pestalozzi B.C., Schanz U., Seebach J.D. (2010). Hyperlipidemic Myeloma: Review of 53 Cases. Ann. Hematol..

[71] Ilyas U., Umar Z., Pansuriya A.M., Mahmood A., Lopez R. (2022). Multiple Myeloma with Retroperitoneal Extramedullary Plasmacytoma Causing Renal Failure and Obstructive Shock From Inferior Vena Cava Compression: A Case Report. Cureus.

[72] Shimokihara K., Kawahara T., Chiba S., Takamoto D., Yao M., Uemura H. (2018). Extramedullary Plasmacytoma of the Testis: A Case Report. Urol. Case Rep..

[73] Stork M., Sevcikova S., Minarik J., Krhovska P., Radocha J., Pospisilova L., Brozova L., Jarkovsky J., Spicka I., Straub J. (2022). Identification of Patients at High Risk of Secondary Extramedullary Multiple Myeloma Development. Br. J. Haematol..

[74] Lazaris V., Hatziri A., Symeonidis A., Kypreos K.E. (2021). The Lipoprotein Transport System in the Pathogenesis of Multiple Myeloma: Advances and Challenges. Front. Oncol..

[75] Farrell M., Fairfield H., Karam M., D’Amico A., Murphy C.S., Falank C., Pistofidi R.S., Cao A., Marinac C.R., Dragon J.A. (2023). Targeting the Fatty Acid Binding Proteins Disrupts Multiple Myeloma Cell Cycle Progression and MYC Signaling. eLife.

[76] Kobari L., Auclair M., Piau O., Ferrand N., Zaoui M., Delhommeau F., Fève B., Sabbah M., Garderet L. (2022). Circulating Cytokines Present in Multiple Myeloma Patients Inhibit the Osteoblastic Differentiation of Adipose Stem Cells. Leukemia.

[77] Ponvilawan B., Charoenngam N., Rittiphairoj T., Ungprasert P. (2020). Receipt of Statins Is Associated with Lower Risk of Multiple Myeloma: Systematic Review and Meta-Analysis. Clin. Lymphoma Myeloma Leuk..

[78] Brånvall E., Ekberg S., Eloranta S., Wästerlid T., Birmann B.M., Smedby K.E. (2020). Statin Use Is Associated with Improved Survival in Multiple Myeloma: A Swedish Population-Based Study of 4315 Patients. Am. J. Hematol..

[79] Gohlke B., Zincke F., Eckert A., Kobelt D., Preissner S., Liebeskind J.M., Gunkel N., Putzker K., Lewis J., Preissner S. (2022). Real-world Evidence for Preventive Effects of Statins on Cancer Incidence: A Trans-Atlantic Analysis. Clin. Transl. Med..

[80] Juneja M., Kobelt D., Walther W., Voss C., Smith J., Specker E., Neuenschwander M., Gohlke B.-O., Dahlmann M., Radetzki S. (2017). Statin and Rottlerin Small-Molecule Inhibitors Restrict Colon Cancer Progression and Metastasis via MACC1. PLoS Biol..

[81] Lonial S., Lee H.C., Badros A., Trudel S., Nooka A.K., Chari A., Abdallah A., Callander N., Sborov D., Suvannasankha A. (2021). Longer Term Outcomes with Single-agent Belantamab Mafodotin in Patients with Relapsed or Refractory Multiple Myeloma: 13-month Follow-up from the Pivotal DREAMM-2 Study. Cancer.

[82] Rosiñol L., Beksac M., Zamagni E., Van de Donk N.W.C.J., Anderson K.C., Badros A., Caers J., Cavo M., Dimopoulos M.-A., Dispenzieri A. (2021). Expert Review on Soft-Tissue Plasmacytomas in Multiple Myeloma: Definition, Disease Assessment and Treatment Considerations. Br. J. Haematol..

[83] Jelinek T., Sevcikova T., Zihala D., Popkova T., Kapustova V., Broskevicova L., Capkova L., Rihova L., Bezdekova R., Sevcikova S. (2022). Limited Efficacy of Daratumumab in Multiple Myeloma with Extramedullary Disease. Leukemia.

[84] Li W., Liu M., Yuan T., Yan L., Cui R., Deng Q. (2022). Efficacy and Follow-up of Humanized Anti-BCMA CAR-T Cell Therapy in Relapsed/Refractory Multiple Myeloma Patients with Extramedullary-Extraosseous, Extramedullary-Bone Related, and without Extramedullary Disease. Hematol. Oncol..

[85] Ho M., Xiao A., Yi D., Zanwar S., Bianchi G. (2022). Treating Multiple Myeloma in the Context of the Bone Marrow Microenvironment. Curr. Oncol..

[86] Galli M., Chatterjee M., Grasso M., Specchia G., Magen H., Einsele H., Celeghini I., Barbieri P., Paoletti D., Pace S. (2018). Phase I Study of the Heparanase Inhibitor Roneparstat: An Innovative Approach for Ultiple Myeloma Therapy. Haematologica.

[87] Federico C., Alhallak K., Sun J., Duncan K., Azab F., Sudlow G.P., de la Puente P., Muz B., Kapoor V., Zhang L. (2020). Tumor Microenvironment-Targeted Nanoparticles Loaded with Bortezomib and ROCK Inhibitor Improve Efficacy in Multiple Myeloma. Nat. Commun..

[88] Sidiqi M.H., Al Saleh A.S., Kumar S.K., Leung N., Jevremovic D., Muchtar E., Gonsalves W.I., Kourelis T.V., Warsame R., Buadi F.K. (2021). Venetoclax for the Treatment of Multiple Myeloma: Outcomes Outside of Clinical Trials. Am. J. Hematol..

[89] Ludwig L.M., Maxcy K.L., LaBelle J.L. (2019). Flow Cytometry-Based Detection and Analysis of BCL-2 Family Proteins and Mitochondrial Outer Membrane Permeabilization (MOMP). Methods Mol. Biol..

[90] Ackler S., Mitten M.J., Foster K., Oleksijew A., Refici M., Tahir S.K., Xiao Y., Tse C., Frost D.J., Fesik S.W. (2010). The Bcl-2 Inhibitor ABT-263 Enhances the Response of Multiple Chemotherapeutic Regimens in Hematologic Tumors in Vivo. Cancer Chemother. Pharmacol..

[91] Yee A.J., Huff C.A., Chari A., Vogl D.T., Gavriatopoulou M., Nooka A.K., Moreau P., Dingli D., Cole C.E., Lonial S. (2019). Response to Therapy and the Effectiveness of Treatment with Selinexor and Dexamethasone in Patients with Penta-Exposed Triple-Class Refractory Myeloma Who Had Plasmacytomas. Blood.

[92] Bahlis N.J., Sutherland H., White D., Sebag M., Lentzsch S., Kotb R., Venner C.P., Gasparetto C., Del Col A., Neri P. (2018). Selinexor plus Low-Dose Bortezomib and Dexamethasone for Patients with Relapsed or Refractory Multiple Myeloma. Blood.

[93] Richardson P.G., Oriol A., Larocca A., Bladé J., Cavo M., Rodriguez-Otero P., Leleu X., Nadeem O., Hiemenz J.W., Hassoun H. (2021). Melflufen and Dexamethasone in Heavily Pretreated Relapsed and Refractory Multiple Myeloma. J. Clin. Oncol..

[94] Miettinen J.J., Kumari R., Traustadottir G.A., Huppunen M.-E., Sergeev P., Majumder M.M., Schepsky A., Gudjonsson T., Lievonen J., Bazou D. (2021). Aminopeptidase Expression in Multiple Myeloma Associates with Disease Progression and Sensitivity to Melflufen. Cancers.

[95] Sriskandarajah P., De Haven Brandon A., MacLeod K., Carragher N.O., Kirkin V., Kaiser M., Whittaker S.R. (2020). Combined Targeting of MEK and the Glucocorticoid Receptor for the Treatment of RAS-Mutant Multiple Myeloma. BMC Cancer.

[96] Gupta S., Master S., Graham C. (2022). Extramedullary Multiple Myeloma: A Patient-Focused Review of the Pathogenesis of Bone Marrow Escape. World J. Oncol..

[97] Dahl I.M.S., Rasmussen T., Kauric G., Husebekk A. (2002). Differential Expression of CD56 and CD44 in the Evolution of Extramedullary Myeloma. Br. J. Haematol..

[98] Mey U.J.M., Renner C., von Moos R. (2017). Vemurafenib in Combination with Cobimetinib in Relapsed and Refractory Extramedullary Multiple Myeloma Harboring the BRAF V600E Mutation. Hematol. Oncol..

[99] Giesen N., Chatterjee M., Scheid C., Poos A.M., Besemer B., Miah K., Benner A., Becker N., Moehler T., Metzler I. (2023). A Phase 2 Clinical Trial of Combined BRAF/MEK Inhibition for BRAFV600E-Mutated Multiple Myeloma. Blood.

[100] Mariño K.V., Cagnoni A.J., Croci D.O., Rabinovich G.A. (2023). Targeting Galectin-Driven Regulatory Circuits in Cancer and Fibrosis. Nat. Rev. Drug Discov..

[101] Storti P., Marchica V., Giuliani N. (2017). Role of Galectins in Multiple Myeloma. Int. J. Mol. Sci..

[102] Storti P., Marchica V., Airoldi I., Donofrio G., Fiorini E., Ferri V., Guasco D., Todoerti K., Silbermann R., Anderson J.L. (2016). Galectin-1 Suppression Delineates a New Strategy to Inhibit Myeloma-Induced Angiogenesis and Tumoral Growth in Vivo. Leukemia.

[103] Cutler C., Lee S.J., Arai S., Rotta M., Zoghi B., Lazaryan A., Ramakrishnan A., DeFilipp Z., Salhotra A., Chai-Ho W. (2021). Belumosudil for Chronic Graft-versus-Host Disease after 2 or More Prior Lines of Therapy: The ROCKstar Study. Blood.

[104] Kalra J., Warburton C., Fang K., Edwards L., Daynard T., Waterhouse D., Dragowska W., Sutherland B.W., Dedhar S., Gelmon K. (2009). QLT0267, a Small Molecule Inhibitor Targeting Integrin-Linked Kinase (ILK), and Docetaxel Can Combine to Produce Synergistic Interactions Linked to Enhanced Cytotoxicity, Reductions in P-AKT Levels, Altered F-Actin Architecture and Improved Treatment Outcomes in an Orthotopic Breast Cancer Model. Breast Cancer Res..

[105] García-Marín J., Rodríguez-Puyol D., Vaquero J.J. (2022). Insight into the Mechanism of Molecular Recognition between Human Integrin-Linked Kinase and Cpd22 and Its Implication at Atomic Level. J. Comput. Aided Mol. Des..

