# Supplementary material for: Proteomic and Metabolomic Analysis of Bone Marrow and Plasma from Patients with Extramedullary Multiple Myeloma Identifies Distinct Protein and Metabolite Signatures
Source: Cancers (Basel). 2023 Jul 25;15(15):3764. doi: 10.3390/cancers15153764 (PMC10417544; doi:10.3390/cancers15153764)
Supplement: Supplementary file 1 [file cancers-15-03764-s001.zip › cancers-2517044-supplementary.pdf]

Supplementary Materials: Proteomic and Metabolomic Analysis of Bone Marrow and Plasma from Patients with Extramedullary Multiple Myeloma Identifies Distinct Protein and Metabolite Signatures

Katie Dunphy, Despina Bazou, Michael Henry, Paula Meleady, Juho J. Miettinen, Caroline A. Heckman, Paul Dowling and Peter O'Gorman

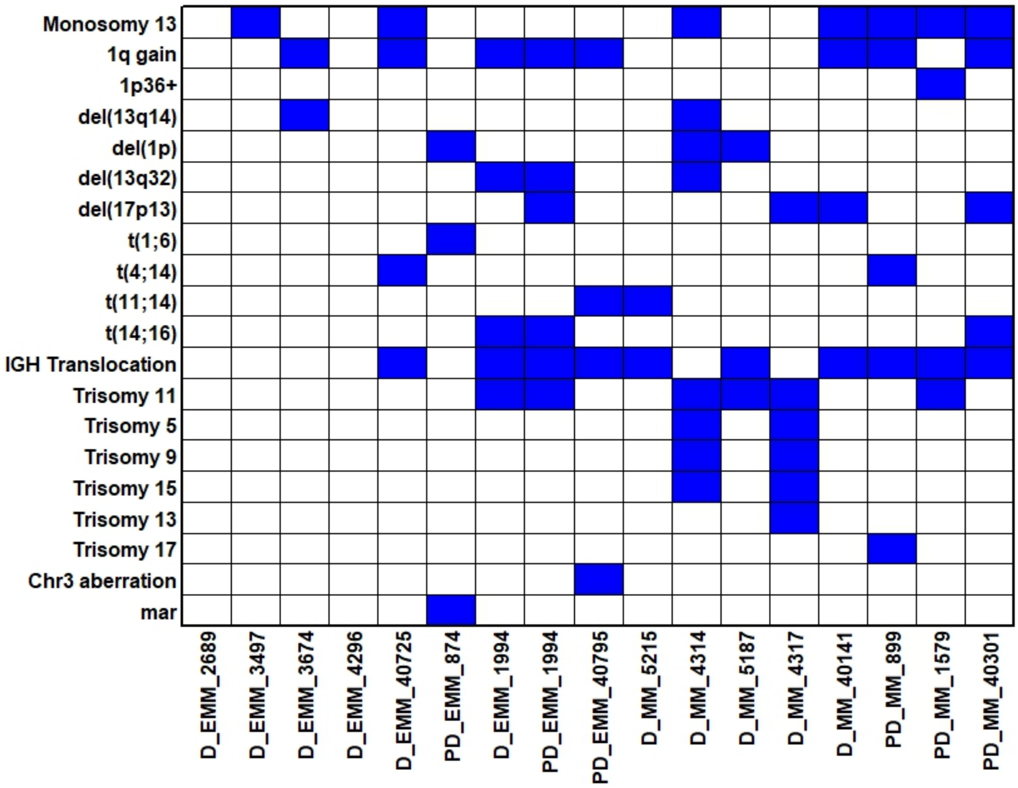

Figure S1. Cytogenetics of patient cohort.

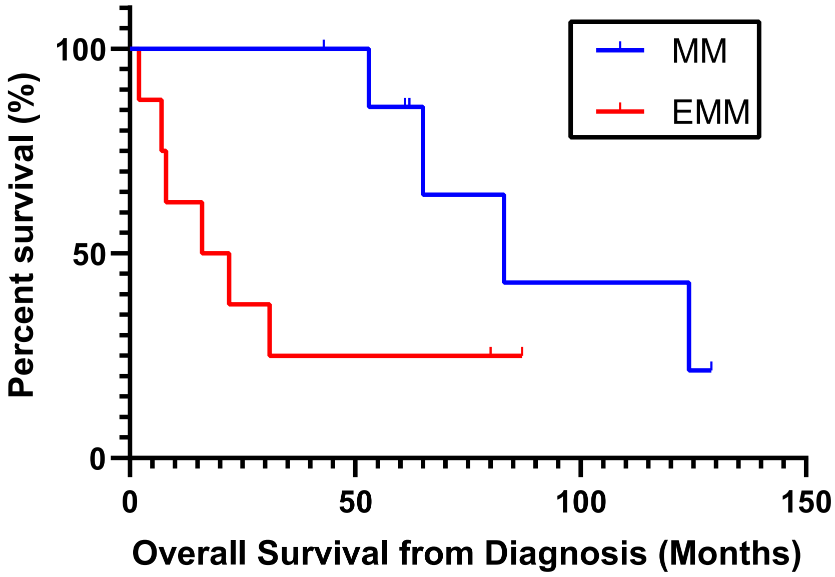

Figure S2. Survival graph illustrating the difference in OS between the EMM group (n=8) and medullary MM group (n=8).

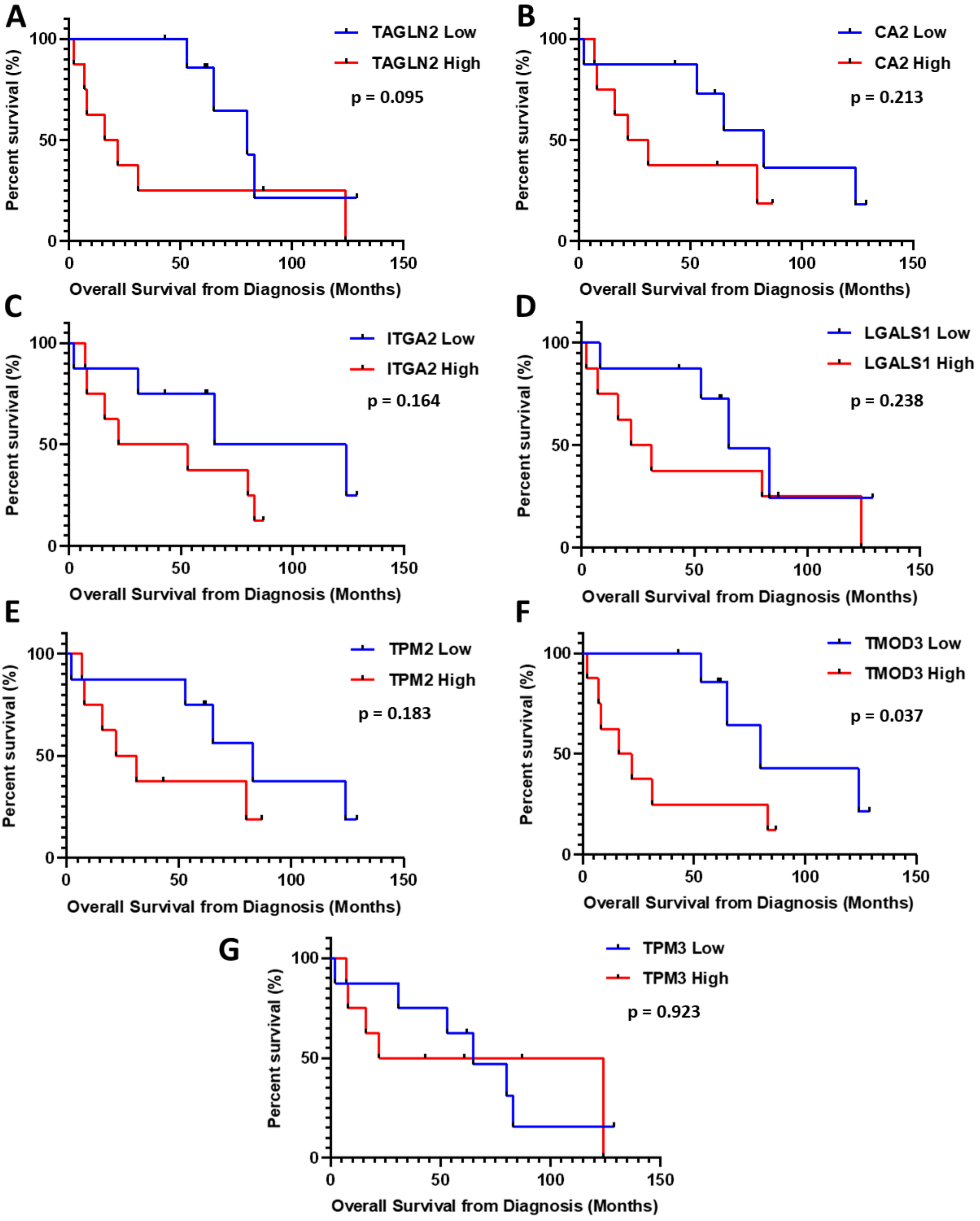

Figure S3: Survival graphs illustrating the difference in OS between patients with high expression and low expression of the seven proteins identified as potential prognostic biomarkers in the CoMMpass dataset. Samples were divided based on median expression levels. (**A**) TAGLN2 (**B**) CA2 (**C**) ITGA2 (**D**) LGALS1 (**E**) TPM2 (**F**) TMOD3 (**G**) TPM3.

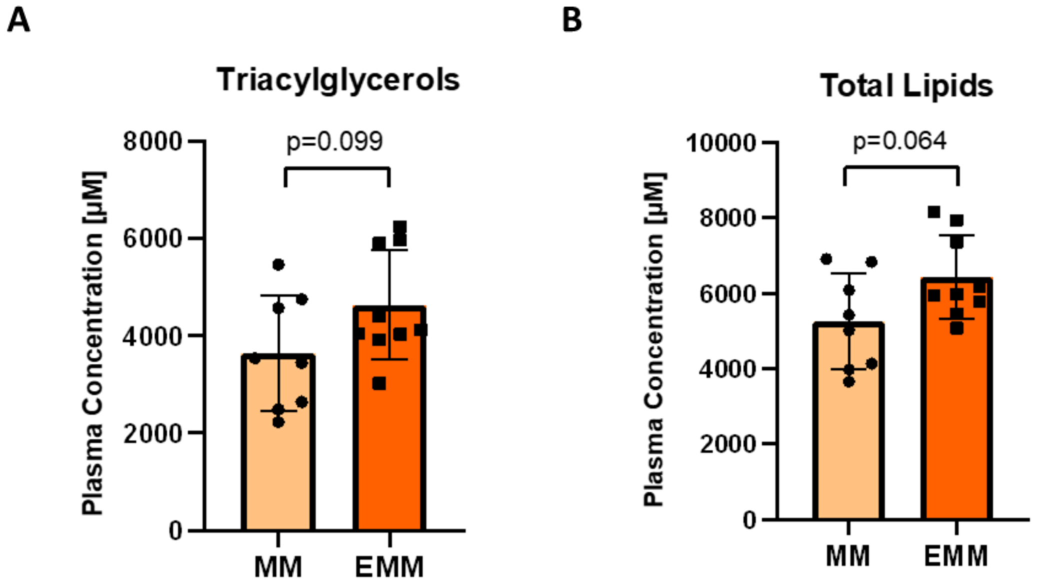

Figure S4. Targeted metabolomics analysis shows a trend towards increased triglycerides and lipids in the plasma of EMM patients (**A**) Total plasma triglyceride concentration in MM patients with and without extramedullary spread. (**B**) Total lipid concentration in MM patients with and without extramedullary spread.

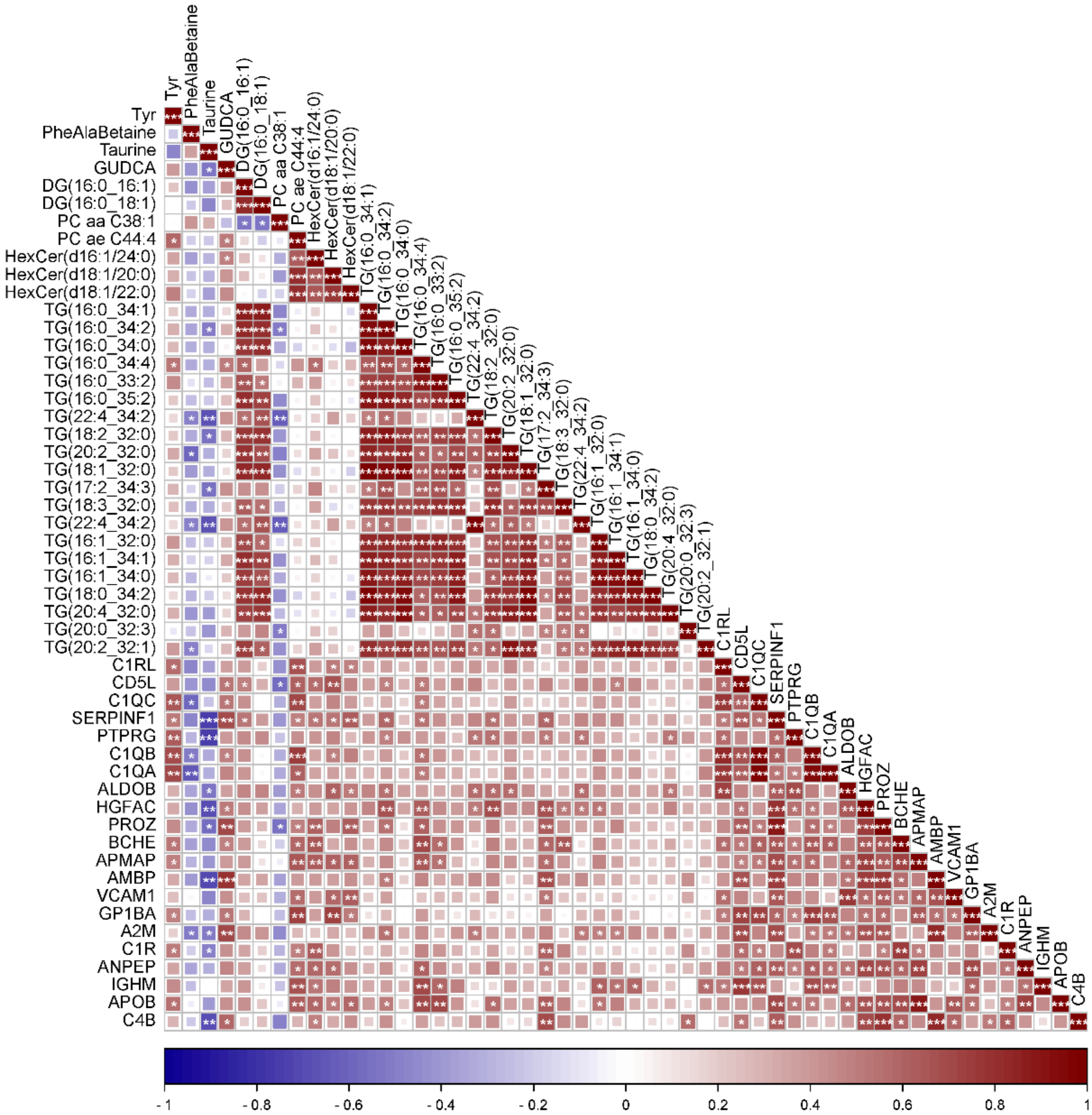

Figure S5. Spearmans correlation matrix between differential metabolites and proteins in plasma. Blue colour indicates negative correlation; Red colour indicates positive correlation. Significant correlations regions were marked by stars (*P < 0.05, **P < 0.01, ***P < 0.001).

Table S1. Full list of statistically significant differentially abundant (SSDA) proteins in EMM bone marrow mononuclear cells (BMNCs) and MM BMNCs.

Protein Accession	Gene Name	Peptide Count	Anova (p)	Log2 Fold Change
P00918	CA2	2	0.0001	2.14
Q8NBJ5	COLGALT1	3	0.0003	0.82
P22087	FBL	2	0.0003	-0.73
P09382	LGALS1	2	0.0005	0.95
Q5JRX3	PITRM1	2	0.0006	1.61
P37802	TAGLN2	8	0.0006	2.22
P16402	H1-3	2	0.0007	-1.53
Q8NBS9	TXNDC5	9	0.0008	-2.11
P17301	ITGA2	2	0.0009	5.13
Q86WV6	STING1	2	0.0009	1.60
Q32MZ4	LRRFIP1	2	0.0009	1.09
Q15833	STXBP2	3	0.0011	1.17
P62328	TMSB4X	3	0.0011	3.11
Q99798	ACO2	3	0.0012	-0.69
Q9NSE4	IARS2	3	0.0014	-1.15
Q9Y320	TMX2	2	0.0014	-2.57
Q13263	TRIM28	2	0.0015	-1.13
P08567	PLEK	4	0.0015	2.45
P30837	ALDH1B1	2	0.0015	-3.03
Q9BY50	SEC11C	4	0.0016	-2.38
Q9UGT4	SUSD2	2	0.0017	5.74
O60610	DIAPH1	5	0.0018	1.15
P08758	ANXA5	2	0.0018	2.94
P07951	TPM2	2	0.0019	1.79
Q7LDG7	RASGRP2	3	0.0019	1.83
Q14019	COTL1	5	0.0020	1.25
P18054	ALOX12	2	0.0020	4.73
Q9NYL9	TMOD3	4	0.0020	1.54
Q13813	SPTAN1	13	0.0023	-1.07
P63000	RAC1	2	0.0025	1.45
P37840	SNCA	3	0.0025	4.21
Q9HBI1	PARVB	5	0.0026	3.60
P18206	VCL	43	0.0028	2.53
Q15942	ZYX	12	0.0029	2.30
P06753	TPM3	5	0.0030	1.05
P07339	CTSD	5	0.0032	0.65
Q3SY69	ALDH1L2	3	0.0033	-2.87
P08240	SRPRA	2	0.0035	-1.31
P30044	PRDX5	2	0.0037	-0.83
P48426	PIP4K2A	5	0.0039	1.22
Q9Y490	TLN1	79	0.0041	2.29
Q7KZF4	SND1	4	0.0042	-1.54
Q15661	TPSAB1	2	0.0043	10.23
P49257	LMAN1	9	0.0043	-1.29
Q9Y4P3	TBL2	3	0.0045	-2.18
P09874	PARP1	5	0.0047	-1.42
P43405	SYK	2	0.0049	2.13
P50552	VASP	4	0.0050	1.61

P67936	TPM4	12	0.0052	1.92
Q01105	SET	2	0.0054	-1.72
Q7L576	CYFIP1	5	0.0054	1.24
Q92506	HSD17B8	2	0.0054	-1.75
Q04917	YWHAH	4	0.0054	0.79
P12235	SLC25A4	4	0.0055	-2.15
O15117	FYB1	2	0.0055	2.55
Q13310	PABPC4	3	0.0056	-2.16
O00429	DNM1L	2	0.0057	2.76
P53992	SEC24C	2	0.0057	-5.27
Q16706	MAN2A1	7	0.0058	-2.69
Q01082	SPTBN1	14	0.0060	-0.97
Q86UX7	FERMT3	14	0.0064	1.65
P48059	LIMS1	4	0.0065	2.52
P42704	LRPPRC	3	0.0066	1.58
O15269	SPTLC1	2	0.0068	0.66
P50502	ST13	3	0.0069	0.61
P00387	CYB5R3	2	0.0070	0.74
O75116	ROCK2	2	0.0070	2.86
P54886	ALDH18A1	3	0.0072	-3.36
Q9Y678	COPG1	4	0.0073	-1.08
P53999	SUB1	6	0.0074	-1.62
P13667	PDIA4	3	0.0074	-1.31
P68032	ACTC1	2	0.0075	1.24
Q9Y251	HPSE	3	0.0077	1.79
Q9UHQ4	BCAP29	2	0.0079	0.99
Q13418	ILK	7	0.0080	2.25
P08236	GUSB	6	0.0080	-1.31
Q15005	SPCS2	3	0.0081	-1.24
P14618	PKM	8	0.0082	0.90
P78417	GSTO1	5	0.0082	1.28
O75083	WDR1	9	0.0083	1.29
P22314	UBA1	2	0.0085	-7.27
Q15404	RSU1	5	0.0085	1.96
P02042	HBD	2	0.0087	0.72
Q15257	PTPA	2	0.0087	0.86
O00299	CLIC1	7	0.0089	0.96
P35555	FBN1	3	0.0091	-5.71
Q96C24	SYTL4	2	0.0095	3.18
Q00610	CLTC	3	0.0099	8.55
P30041	PRDX6	2	0.0100	1.74
P63104	YWHAZ	13	0.0102	0.88
P22626	HNRNPA2B1	5	0.0102	-0.61
P29350	PTPN6	2	0.0103	1.44
P61224	RAP1B	5	0.0104	1.70
P17858	PFKL	4	0.0105	-0.88
P01011	SERPINA3	10	0.0105	1.10
P21333	FLNA	67	0.0106	1.41
Q16822	PCK2	2	0.0108	-2.28
P00488	F13A1	10	0.0110	2.05
P04843	RPN1	2	0.0112	-0.69

P69905	HBA1	3	0.0112	1.01
Q9UHG3	PCYOX1	4	0.0112	-0.83
Q9H299	SH3BGRL3	4	0.0115	1.14
P02649	APOE	2	0.0118	1.10
P51991	HNRNPA3	3	0.0120	-0.61
Q9BWS9	CHID1	2	0.0121	-1.56
O60832	DKC1	2	0.0123	-0.97
Q9P2E9	RRBP1	5	0.0123	-2.85
P49736	MCM2	2	0.0123	1.23
P18827	SDC1	2	0.0128	-5.89
Q92619	ARHGAP45	2	0.0128	0.97
P00450	CP	6	0.0132	1.71
Q01813	PFKP	2	0.0134	1.85
P08519	LPA	11	0.0134	0.97
P60709	ACTB	17	0.0135	0.95
P07195	LDHB	3	0.0136	1.05
Q13162	PRDX4	8	0.0140	-1.00
P35579	MYH9	50	0.0140	0.92
Q9NZN3	EHD3	4	0.0141	2.66
Q6UW68	TMEM205	2	0.0141	-0.66
Q9H2U2	PPA2	2	0.0141	-0.71
O95810	CAVIN2	8	0.0142	3.26
P14770	GP9	3	0.0143	1.73
O00567	NOP56	2	0.0145	-0.79
P04075	ALDOA	3	0.0149	1.25
Q9Y2Q0	ATP8A1	2	0.0149	1.23
P68363	TUBA1B	7	0.0156	0.70
Q8WU39	MZB1	3	0.0158	-1.67
Q96I99	SUCLG2	3	0.0159	-0.63
Q13094	LCP2	2	0.0161	3.83
Q6P2Q9	PRPF8	2	0.0167	-1.04
P21291	CSRP1	3	0.0171	2.12
P52566	ARHGDIB	4	0.0176	0.78
Q01518	CAP1	7	0.0176	0.70
Q9UKM9	RALY	2	0.0179	-0.69
P02654	APOC1	2	0.0183	0.77
P17987	TCP1	2	0.0185	1.14
P21796	VDAC1	2	0.0187	-0.69
P42126	ECI1	2	0.0187	-1.03
Q07065	CKAP4	12	0.0191	-0.75
Q9UBW5	BIN2	5	0.0192	1.06
Q8N6Q3	CD177	2	0.0194	-2.65
O43488	AKR7A2	2	0.0195	1.04
Q09666	AHNAK	6	0.0198	0.71
Q9Y2X3	NOP58	2	0.0199	-1.12
P06702	S100A9	5	0.0204	-0.74
Q3ZCW2	LGALSL	2	0.0208	2.40
P55072	VCP	5	0.0213	-0.70
O00151	PDLIM1	3	0.0219	2.66
Q03252	LMNB2	6	0.0223	-0.69
P36542	ATP5F1C	2	0.0226	-2.02

P30085	CMPK1	2	0.0229	1.26
P13224	GP1BB	3	0.0229	1.53
P05556	ITGB1	3	0.0231	1.17
O75477	ERLIN1	2	0.0234	0.69
P04439	HLA-A	2	0.0239	1.22
O00506	STK25	3	0.0241	1.19
P02794	FTH1	2	0.0241	0.80
Q9ULV4	CORO1C	6	0.0243	1.67
P12111	COL6A3	2	0.0244	-2.83
P02788	LTF	8	0.0244	-1.01
P00491	PNP	2	0.0247	1.37
P16109	SELP	3	0.0248	1.97
P05109	S100A8	4	0.0249	-0.77
Q92520	FAM3C	2	0.0250	-1.50
Q9H4B7	TUBB1	3	0.0251	2.53
P53618	COPB1	3	0.0252	-0.59
P39656	DDOST	2	0.0252	-0.83
P12259	F5	10	0.0252	1.46
P16671	CD36	4	0.0252	1.48
Q02218	OGDH	3	0.0252	-0.77
Q9Y3F4	STRAP	2	0.0255	1.12
P61353	RPL27	2	0.0257	-0.69
O95866	MPIG6B	2	0.0257	2.80
P50213	IDH3A	2	0.0264	-1.14
P16949	STMN1	2	0.0265	1.04
P53396	ACLY	2	0.0268	0.63
P35749	MYH11	6	0.0268	0.79
P0DP25	CALM3	2	0.0270	0.78
P0DMV9	HSPA1B	2	0.0271	1.86
P06576	ATP5F1B	2	0.0272	-5.29
P02775	PPBP	2	0.0277	1.86
O43772	SLC25A20	2	0.0283	1.73
P05106	ITGB3	15	0.0283	2.03
P08514	ITGA2B	13	0.0283	1.74
P04275	VWF	2	0.0284	-2.55
O76074	PDE5A	2	0.0284	1.77
Q0VD83	APOBR	2	0.0284	1.27
P07203	GPX1	2	0.0286	0.66
P07359	GP1BA	6	0.0288	2.15
Q00341	HDLBP	2	0.0289	-3.38
Q13586	STIM1	3	0.0290	1.17
P14625	HSP90B1	6	0.0298	-1.07
P60981	DSTN	2	0.0304	1.83
P12814	ACTN1	10	0.0306	1.16
Q9HCC0	MCCC2	2	0.0306	-3.35
Q1KMD3	HNRNPUL2	2	0.0308	-0.74
P16284	PECAM1	3	0.0308	0.93
P23528	CFL1	3	0.0308	0.94
P02652	APOA2	2	0.0319	10.06
O95352	ATG7	2	0.0319	-1.54
P07996	THBS1	16	0.0321	1.68

P13010	XRCC5	2	0.0324	-1.36
P27105	STOM	2	0.0328	0.70
P41240	CSK	2	0.0331	0.81
P16615	ATP2A2	2	0.0331	0.61
B5ME19	EIF3CL	3	0.0342	-0.94
Q99439	CNN2	2	0.0356	0.90
P60660	MYL6	3	0.0357	0.66
O75643	SNRNP200	3	0.0362	-0.99
Q9UHL4	DPP7	2	0.0375	-1.07
P30405	PPIF	2	0.0376	0.95
P01137	TGFB1	2	0.0388	1.24
P12931	SRC	3	0.0389	1.71
P24752	ACAT1	3	0.0392	-1.07
P31040	SDHA	2	0.0396	-0.77
P00505	GOT2	2	0.0397	-0.73
P35606	COPB2	5	0.0401	-0.77
P07737	PFN1	3	0.0402	0.61
Q9H0U4	RAB1B	2	0.0406	1.33
Q86VP6	CAND1	2	0.0420	3.21
Q07955	SRSF1	2	0.0420	-0.60
P68871	HBB	3	0.0424	0.73
O14980	XPO1	2	0.0454	-0.98
Q13201	MMRN1	2	0.0467	1.23
P31146	CORO1A	2	0.0484	-0.91

Table S2. Metabolites with signifcant differential abundance in the plasma of MM and EMM patients. Statistically significantly differentially abundant metabolites ($p > 0.05$, FC > 1.2 or < 0.833, VIP > 1).

Metabolite	Compound Class	Fold Change (EMM/MM)	VIP	P-value
TG(22:4_32:0)	Triacylglycerol	2.341	2.500	0.001
TG(16:0_34:2)	Triacylglycerol	1.701	2.301	0.003
HexCer(d18:1/20:0)	Hexosylceramide	1.732	2.261	0.004
TG(18:2_32:0)	Triacylglycerol	1.658	2.252	0.005
Taurine	Amino acid - related	0.547	2.235	0.006
TG(16:0_34:1)	Triacylglycerol	1.813	2.120	0.008
TG(20:2_32:0)	Triacylglycerol	1.860	2.145	0.008
HexCer(d16:1/24:0)	Hexosylceramide	1.843	2.124	0.009
PC aa C38:1	Phosphatidylcholine	0.486	1.989	0.011
TG(18:1_32:0)	Triacylglycerol	1.680	1.987	0.014
TG(22:4_34:2)	Triacylglycerol	1.534	1.971	0.015
DG(16:0_16:1)	Diacylglycerol	1.743	1.915	0.018
TG(18:3_32:0)	Triacylglycerol	1.605	1.921	0.020
TG(17:2_34:3)	Triacylglycerol	1.818	1.761	0.026
TG(16:1_32:0)	Triacylglycerol	2.429	1.839	0.027
TG(16:1_34:1)	Triacylglycerol	1.552	1.808	0.028
PheAlaBetaine	Amino acid - related	0.673	1.838	0.030
DG(16:0_18:1)	Diacylglycerol	1.481	1.800	0.031
TG(16:0_34:0)	Triacylglycerol	1.845	1.781	0.033
TG(16:0_34:4)	Triacylglycerol	1.472	1.822	0.033
TG(16:1_34:0)	Triacylglycerol	1.693	1.750	0.034
TG(18:0_34:2)	Triacylglycerol	1.448	1.752	0.036

TG(20:4_32:0)	Triacylglycerol	2.010	1.752	0.037
GUDCA	Bile acid	3.623	1.855	0.037
HexCer(d18:1/22:0)	Hexosylceramide	1.437	1.820	0.037
PC ae C44:4	Phosphatidylcholine	1.489	1.769	0.041
TG(16:0_35:2)	Triacylglycerol	1.377	1.726	0.042
TG(16:0_33:2)	Triacylglycerol	1.499	1.769	0.043
TG(20:2_32:1)	Triacylglycerol	1.389	1.706	0.044
TG(20:0_32:3)	Triacylglycerol	1.528	1.559	0.046
Tyr	Amino acid	1.432	1.815	0.048
